# Measurements of the associated production of a Z boson and b jets in pp collisions at ${\sqrt{s}} = 8\,\text {TeV}$

**DOI:** 10.1140/epjc/s10052-017-5140-y

**Published:** 2017-11-08

**Authors:** V. Khachatryan, A. M. Sirunyan, A. Tumasyan, W. Adam, E. Asilar, T. Bergauer, J. Brandstetter, E. Brondolin, M. Dragicevic, J. Erö, M. Flechl, M. Friedl, R. Frühwirth, V. M. Ghete, C. Hartl, N. Hörmann, J. Hrubec, M. Jeitler, A. König, M. Krammer, I. Krätschmer, D. Liko, T. Matsushita, I. Mikulec, D. Rabady, N. Rad, B. Rahbaran, H. Rohringer, J. Schieck, J. Strauss, W. Treberer-Treberspurg, W. Waltenberger, C.-E. Wulz, V. Mossolov, N. Shumeiko, J. Suarez Gonzalez, S. Alderweireldt, T. Cornelis, E. A. De Wolf, X. Janssen, A. Knutsson, J. Lauwers, S. Luyckx, M. Van De Klundert, H. Van Haevermaet, P. Van Mechelen, N. Van Remortel, A. Van Spilbeeck, S. Abu Zeid, F. Blekman, J. D’Hondt, N. Daci, I. De Bruyn, K. Deroover, N. Heracleous, J. Keaveney, S. Lowette, S. Moortgat, L. Moreels, A. Olbrechts, Q. Python, D. Strom, S. Tavernier, W. Van Doninck, P. Van Mulders, I. Van Parijs, H. Brun, C. Caillol, B. Clerbaux, G. De Lentdecker, G. Fasanella, L. Favart, R. Goldouzian, A. Grebenyuk, G. Karapostoli, T. Lenzi, A. Léonard, T. Maerschalk, A. Marinov, A. Randle-conde, T. Seva, C. Vander Velde, P. Vanlaer, R. Yonamine, F. Zenoni, F. Zhang, L. Benucci, A. Cimmino, S. Crucy, D. Dobur, A. Fagot, G. Garcia, M. Gul, J. Mccartin, A. A. Ocampo Rios, D. Poyraz, D. Ryckbosch, S. Salva, R. Schöfbeck, M. Sigamani, M. Tytgat, W. Van Driessche, E. Yazgan, N. Zaganidis, C. Beluffi, O. Bondu, S. Brochet, G. Bruno, A. Caudron, L. Ceard, S. De Visscher, C. Delaere, M. Delcourt, L. Forthomme, B. Francois, A. Giammanco, A. Jafari, P. Jez, M. Komm, V. Lemaitre, A. Magitteri, A. Mertens, M. Musich, C. Nuttens, K. Piotrzkowski, L. Quertenmont, M. Selvaggi, M. Vidal Marono, S. Wertz, N. Beliy, G. H. Hammad, W. L. Aldá Júnior, F. L. Alves, G. A. Alves, L. Brito, M. Correa Martins Junior, M. Hamer, C. Hensel, A. Moraes, M. E. Pol, P. Rebello Teles, E. Belchior Batista Das Chagas, W. Carvalho, J. Chinellato, A. Custódio, E. M. Da Costa, D. De Jesus Damiao, C. De Oliveira Martins, S. Fonseca De Souza, L. M. Huertas Guativa, H. Malbouisson, D. Matos Figueiredo, C. Mora Herrera, L. Mundim, H. Nogima, W. L. Prado Da Silva, A. Santoro, A. Sznajder, E. J. Tonelli Manganote, A. Vilela Pereira, S. Ahuja, C. A. Bernardes, A. De Souza Santos, S. Dogra, T. R. Fernandez Perez Tomei, E. M. Gregores, P. G. Mercadante, C. S. Moon, S. F. Novaes, Sandra S. Padula, D. Romero Abad, J. C. Ruiz Vargas, A. Aleksandrov, R. Hadjiiska, P. Iaydjiev, M. Rodozov, S. Stoykova, G. Sultanov, M. Vutova, A. Dimitrov, I. Glushkov, L. Litov, B. Pavlov, P. Petkov, W. Fang, M. Ahmad, J. G. Bian, G. M. Chen, H. S. Chen, M. Chen, T. Cheng, R. Du, C. H. Jiang, D. Leggat, R. Plestina, F. Romeo, S. M. Shaheen, A. Spiezia, J. Tao, C. Wang, Z. Wang, H. Zhang, C. Asawatangtrakuldee, Y. Ban, Q. Li, S. Liu, Y. Mao, S. J. Qian, D. Wang, Z. Xu, C. Avila, A. Cabrera, L. F. Chaparro Sierra, C. Florez, J. P. Gomez, B. Gomez Moreno, J. C. Sanabria, N. Godinovic, D. Lelas, I. Puljak, P. M. Ribeiro Cipriano, Z. Antunovic, M. Kovac, V. Brigljevic, D. Ferencek, K. Kadija, J. Luetic, S. Micanovic, L. Sudic, A. Attikis, G. Mavromanolakis, J. Mousa, C. Nicolaou, F. Ptochos, P. A. Razis, H. Rykaczewski, M. Finger, M. Finger Jr., E. Carrera Jarrin, A. A. Abdelalim, E. El-khateeb, T. Elkafrawy, M. A. Mahmoud, B. Calpas, M. Kadastik, M. Murumaa, L. Perrini, M. Raidal, A. Tiko, C. Veelken, P. Eerola, J. Pekkanen, M. Voutilainen, J. Härkönen, V. Karimäki, R. Kinnunen, T. Lampén, K. Lassila-Perini, S. Lehti, T. Lindén, P. Luukka, T. Peltola, J. Tuominiemi, E. Tuovinen, L. Wendland, J. Talvitie, T. Tuuva, M. Besancon, F. Couderc, M. Dejardin, D. Denegri, B. Fabbro, J. L. Faure, C. Favaro, F. Ferri, S. Ganjour, A. Givernaud, P. Gras, G. Hamel de Monchenault, P. Jarry, E. Locci, M. Machet, J. Malcles, J. Rander, A. Rosowsky, M. Titov, A. Zghiche, A. Abdulsalam, I. Antropov, S. Baffioni, F. Beaudette, P. Busson, L. Cadamuro, E. Chapon, C. Charlot, O. Davignon, L. Dobrzynski, R. Granier de Cassagnac, M. Jo, S. Lisniak, P. Miné, I. N. Naranjo, M. Nguyen, C. Ochando, G. Ortona, P. Paganini, P. Pigard, S. Regnard, R. Salerno, Y. Sirois, T. Strebler, Y. Yilmaz, A. Zabi, J.-L. Agram, J. Andrea, A. Aubin, D. Bloch, J.-M. Brom, M. Buttignol, E. C. Chabert, N. Chanon, C. Collard, E. Conte, X. Coubez, J.-C. Fontaine, D. Gelé, U. Goerlach, C. Goetzmann, A.-C. Le Bihan, J. A. Merlin, K. Skovpen, P. Van Hove, S. Gadrat, S. Beauceron, C. Bernet, G. Boudoul, E. Bouvier, C. A. Carrillo Montoya, R. Chierici, D. Contardo, B. Courbon, P. Depasse, H. El Mamouni, J. Fan, J. Fay, S. Gascon, M. Gouzevitch, B. Ille, F. Lagarde, I. B. Laktineh, M. Lethuillier, L. Mirabito, A. L. Pequegnot, S. Perries, A. Popov, J. D. Ruiz Alvarez, D. Sabes, V. Sordini, M. Vander Donckt, P. Verdier, S. Viret, T. Toriashvili, I. Bagaturia, C. Autermann, S. Beranek, L. Feld, A. Heister, M. K. Kiesel, K. Klein, M. Lipinski, A. Ostapchuk, M. Preuten, F. Raupach, S. Schael, C. Schomakers, J. F. Schulte, J. Schulz, T. Verlage, H. Weber, V. Zhukov, M. Ata, M. Brodski, E. Dietz-Laursonn, D. Duchardt, M. Endres, M. Erdmann, S. Erdweg, T. Esch, R. Fischer, A. Güth, T. Hebbeker, C. Heidemann, K. Hoepfner, S. Knutzen, M. Merschmeyer, A. Meyer, P. Millet, S. Mukherjee, M. Olschewski, K. Padeken, P. Papacz, T. Pook, M. Radziej, H. Reithler, M. Rieger, F. Scheuch, L. Sonnenschein, D. Teyssier, S. Thüer, V. Cherepanov, Y. Erdogan, G. Flügge, H. Geenen, M. Geisler, F. Hoehle, B. Kargoll, T. Kress, A. Künsken, J. Lingemann, A. Nehrkorn, A. Nowack, I. M. Nugent, C. Pistone, O. Pooth, A. Stahl, M. Aldaya Martin, I. Asin, K. Beernaert, O. Behnke, U. Behrens, K. Borras, A. Campbell, P. Connor, C. Contreras-Campana, F. Costanza, C. Diez Pardos, G. Dolinska, S. Dooling, G. Eckerlin, D. Eckstein, T. Eichhorn, E. Gallo, J. Garay Garcia, A. Geiser, A. Gizhko, J. M. Grados Luyando, P. Gunnellini, A. Harb, J. Hauk, M. Hempel, H. Jung, A. Kalogeropoulos, O. Karacheban, M. Kasemann, J. Kieseler, C. Kleinwort, I. Korol, W. Lange, A. Lelek, J. Leonard, K. Lipka, A. Lobanov, W. Lohmann, R. Mankel, I.-A. Melzer-Pellmann, A. B. Meyer, G. Mittag, J. Mnich, A. Mussgiller, E. Ntomari, D. Pitzl, R. Placakyte, A. Raspereza, B. Roland, M. Ö. Sahin, P. Saxena, T. Schoerner-Sadenius, C. Seitz, S. Spannagel, N. Stefaniuk, K. D. Trippkewitz, G. P. Van Onsem, R. Walsh, C. Wissing, V. Blobel, M. Centis Vignali, A. R. Draeger, T. Dreyer, J. Erfle, E. Garutti, K. Goebel, D. Gonzalez, M. Görner, J. Haller, M. Hoffmann, R. S. Höing, A. Junkes, R. Klanner, R. Kogler, N. Kovalchuk, T. Lapsien, T. Lenz, I. Marchesini, D. Marconi, M. Meyer, M. Niedziela, D. Nowatschin, J. Ott, F. Pantaleo, T. Peiffer, A. Perieanu, N. Pietsch, J. Poehlsen, C. Sander, C. Scharf, P. Schleper, E. Schlieckau, A. Schmidt, S. Schumann, J. Schwandt, H. Stadie, G. Steinbrück, F. M. Stober, H. Tholen, D. Troendle, E. Usai, L. Vanelderen, A. Vanhoefer, B. Vormwald, C. Barth, C. Baus, J. Berger, C. Böser, E. Butz, T. Chwalek, F. Colombo, W. De Boer, A. Descroix, A. Dierlamm, S. Fink, F. Frensch, R. Friese, M. Giffels, A. Gilbert, D. Haitz, F. Hartmann, S. M. Heindl, U. Husemann, I. Katkov, A. Kornmayer, P. Lobelle Pardo, B. Maier, H. Mildner, M. U. Mozer, T. Müller, Th. Müller, M. Plagge, G. Quast, K. Rabbertz, S. Röcker, F. Roscher, M. Schröder, G. Sieber, H. J. Simonis, R. Ulrich, J. Wagner-Kuhr, S. Wayand, M. Weber, T. Weiler, S. Williamson, C. Wöhrmann, R. Wolf, G. Anagnostou, G. Daskalakis, T. Geralis, V. A. Giakoumopoulou, A. Kyriakis, D. Loukas, A. Psallidas, I. Topsis-Giotis, A. Agapitos, S. Kesisoglou, A. Panagiotou, N. Saoulidou, E. Tziaferi, I. Evangelou, G. Flouris, C. Foudas, P. Kokkas, N. Loukas, N. Manthos, I. Papadopoulos, E. Paradas, J. Strologas, N. Filipovic, G. Bencze, C. Hajdu, P. Hidas, D. Horvath, F. Sikler, V. Veszpremi, G. Vesztergombi, A. J. Zsigmond, N. Beni, S. Czellar, J. Karancsi, J. Molnar, Z. Szillasi, M. Bartók, A. Makovec, P. Raics, Z. L. Trocsanyi, B. Ujvari, S. Choudhury, P. Mal, K. Mandal, A. Nayak, D. K. Sahoo, N. Sahoo, S. K. Swain, S. Bansal, S. B. Beri, V. Bhatnagar, R. Chawla, R. Gupta, U. Bhawandeep, A. K. Kalsi, A. Kaur, M. Kaur, R. Kumar, A. Mehta, M. Mittal, J. B. Singh, G. Walia, Ashok Kumar, A. Bhardwaj, B. C. Choudhary, R. B. Garg, S. Keshri, A. Kumar, S. Malhotra, M. Naimuddin, N. Nishu, K. Ranjan, R. Sharma, V. Sharma, R. Bhattacharya, S. Bhattacharya, K. Chatterjee, S. Dey, S. Dutta, S. Ghosh, N. Majumdar, A. Modak, K. Mondal, S. Mukhopadhyay, S. Nandan, A. Purohit, A. Roy, D. Roy, S. Roy Chowdhury, S. Sarkar, M. Sharan, R. Chudasama, D. Dutta, V. Jha, V. Kumar, A. K. Mohanty, L. M. Pant, P. Shukla, A. Topkar, S. Chauhan, S. Dube, A. Kapoor, K. Kothekar, A. Rane, S. Sharma, H. Bakhshiansohi, H. Behnamian, S. M. Etesami, A. Fahim, M. Khakzad, M. Mohammadi Najafabadi, M. Naseri, S. Paktinat Mehdiabadi, F. Rezaei Hosseinabadi, B. Safarzadeh, M. Zeinali, M. Felcini, M. Grunewald, M. Abbrescia, C. Calabria, C. Caputo, A. Colaleo, D. Creanza, L. Cristella, N. De Filippis, M. De Palma, L. Fiore, G. Iaselli, G. Maggi, M. Maggi, G. Miniello, S. My, S. Nuzzo, A. Pompili, G. Pugliese, R. Radogna, A. Ranieri, G. Selvaggi, L. Silvestris, R. Venditti, G. Abbiendi, C. Battilana, D. Bonacorsi, S. Braibant-Giacomelli, L. Brigliadori, R. Campanini, P. Capiluppi, A. Castro, F. R. Cavallo, S. S. Chhibra, G. Codispoti, M. Cuffiani, G. M. Dallavalle, F. Fabbri, A. Fanfani, D. Fasanella, P. Giacomelli, C. Grandi, L. Guiducci, S. Marcellini, G. Masetti, A. Montanari, F. L. Navarria, A. Perrotta, A. M. Rossi, T. Rovelli, G. P. Siroli, N. Tosi, G. Cappello, M. Chiorboli, S. Costa, A. Di Mattia, F. Giordano, R. Potenza, A. Tricomi, C. Tuve, G. Barbagli, V. Ciulli, C. Civinini, R. D’Alessandro, E. Focardi, V. Gori, P. Lenzi, M. Meschini, S. Paoletti, G. Sguazzoni, L. Viliani, L. Benussi, S. Bianco, F. Fabbri, D. Piccolo, F. Primavera, V. Calvelli, F. Ferro, M. Lo Vetere, M. R. Monge, E. Robutti, S. Tosi, L. Brianza, M. E. Dinardo, S. Fiorendi, S. Gennai, A. Ghezzi, P. Govoni, S. Malvezzi, R. A. Manzoni, B. Marzocchi, D. Menasce, L. Moroni, M. Paganoni, D. Pedrini, S. Pigazzini, S. Ragazzi, N. Redaelli, T. Tabarelli de Fatis, S. Buontempo, N. Cavallo, S. Di Guida, M. Esposito, F. Fabozzi, A. O. M. Iorio, G. Lanza, L. Lista, S. Meola, M. Merola, P. Paolucci, C. Sciacca, F. Thyssen, P. Azzi, N. Bacchetta, L. Benato, D. Bisello, A. Boletti, R. Carlin, P. Checchia, M. Dall’Osso, P. De Castro Manzano, T. Dorigo, U. Dosselli, F. Gasparini, U. Gasparini, A. Gozzelino, K. Kanishchev, S. Lacaprara, M. Margoni, A. T. Meneguzzo, J. Pazzini, N. Pozzobon, P. Ronchese, F. Simonetto, E. Torassa, M. Tosi, S. Vanini, S. Ventura, M. Zanetti, P. Zotto, A. Zucchetta, G. Zumerle, A. Braghieri, A. Magnani, P. Montagna, S. P. Ratti, V. Re, C. Riccardi, P. Salvini, I. Vai, P. Vitulo, L. Alunni Solestizi, G. M. Bilei, D. Ciangottini, L. Fanò, P. Lariccia, R. Leonardi, G. Mantovani, M. Menichelli, A. Saha, A. Santocchia, K. Androsov, P. Azzurri, G. Bagliesi, J. Bernardini, T. Boccali, R. Castaldi, M. A. Ciocci, R. Dell’Orso, S. Donato, G. Fedi, A. Giassi, M. T. Grippo, F. Ligabue, T. Lomtadze, L. Martini, A. Messineo, F. Palla, A. Rizzi, A. Savoy-Navarro, P. Spagnolo, R. Tenchini, G. Tonelli, A. Venturi, P. G. Verdini, L. Barone, F. Cavallari, G. D’imperio, D. Del Re, M. Diemoz, S. Gelli, C. Jorda, E. Longo, F. Margaroli, P. Meridiani, G. Organtini, R. Paramatti, F. Preiato, S. Rahatlou, C. Rovelli, F. Santanastasio, N. Amapane, R. Arcidiacono, S. Argiro, M. Arneodo, N. Bartosik, R. Bellan, C. Biino, N. Cartiglia, M. Costa, R. Covarelli, A. Degano, N. Demaria, L. Finco, B. Kiani, C. Mariotti, S. Maselli, E. Migliore, V. Monaco, E. Monteil, M. M. Obertino, L. Pacher, N. Pastrone, M. Pelliccioni, G. L. Pinna Angioni, F. Ravera, A. Romero, M. Ruspa, R. Sacchi, V. Sola, A. Solano, A. Staiano, P. Traczyk, S. Belforte, V. Candelise, M. Casarsa, F. Cossutti, G. Della Ricca, C. La Licata, A. Schizzi, A. Zanetti, S. K. Nam, D. H. Kim, G. N. Kim, M. S. Kim, D. J. Kong, S. Lee, S. W. Lee, Y. D. Oh, A. Sakharov, D. C. Son, Y. C. Yang, J. A. Brochero Cifuentes, H. Kim, T. J. Kim, S. Song, S. Cho, S. Choi, Y. Go, D. Gyun, B. Hong, Y. Jo, Y. Kim, B. Lee, K. Lee, K. S. Lee, S. Lee, J. Lim, S. K. Park, Y. Roh, H. D. Yoo, M. Choi, H. Kim, H. Kim, J. H. Kim, J. S. H. Lee, I. C. Park, G. Ryu, M. S. Ryu, Y. Choi, J. Goh, D. Kim, E. Kwon, J. Lee, I. Yu, V. Dudenas, A. Juodagalvis, J. Vaitkus, I. Ahmed, Z. A. Ibrahim, J. R. Komaragiri, M. A. B. Md Ali, F. Mohamad Idris, W. A. T. Wan Abdullah, M. N. Yusli, Z. Zolkapli, E. Casimiro Linares, H. Castilla-Valdez, E. De La Cruz-Burelo, I. Heredia-De La Cruz, A. Hernandez-Almada, R. Lopez-Fernandez, J. Mejia Guisao, A. Sanchez-Hernandez, S. Carrillo Moreno, F. Vazquez Valencia, I. Pedraza, H. A. Salazar Ibarguen, C. Uribe Estrada, A. Morelos Pineda, D. Krofcheck, P. H. Butler, A. Ahmad, M. Ahmad, Q. Hassan, H. R. Hoorani, W. A. Khan, T. Khurshid, M. Shoaib, M. Waqas, H. Bialkowska, M. Bluj, B. Boimska, T. Frueboes, M. Górski, M. Kazana, K. Nawrocki, K. Romanowska-Rybinska, M. Szleper, P. Zalewski, G. Brona, K. Bunkowski, A. Byszuk, K. Doroba, A. Kalinowski, M. Konecki, J. Krolikowski, M. Misiura, M. Olszewski, M. Walczak, P. Bargassa, C. Beirão Da Cruz E Silva, A. Di Francesco, P. Faccioli, P. G. Ferreira Parracho, M. Gallinaro, J. Hollar, N. Leonardo, L. Lloret Iglesias, M. V. Nemallapudi, F. Nguyen, J. Rodrigues Antunes, J. Seixas, O. Toldaiev, D. Vadruccio, J. Varela, P. Vischia, S. Afanasiev, I. Golutvin, A. Kamenev, V. Karjavin, V. Korenkov, A. Lanev, A. Malakhov, V. Matveev, V. V. Mitsyn, P. Moisenz, V. Palichik, V. Perelygin, S. Shmatov, S. Shulha, N. Skatchkov, V. Smirnov, E. Tikhonenko, N. Voytishin, A. Zarubin, V. Golovtsov, Y. Ivanov, V. Kim, E. Kuznetsova, P. Levchenko, V. Murzin, V. Oreshkin, I. Smirnov, V. Sulimov, L. Uvarov, S. Vavilov, A. Vorobyev, Yu. Andreev, A. Dermenev, S. Gninenko, N. Golubev, A. Karneyeu, M. Kirsanov, N. Krasnikov, A. Pashenkov, D. Tlisov, A. Toropin, V. Epshteyn, V. Gavrilov, N. Lychkovskaya, V. Popov, I. Pozdnyakov, G. Safronov, A. Spiridonov, M. Toms, E. Vlasov, A. Zhokin, R. Chistov, M. Danilov, O. Markin, E. Popova, V. Rusinov, V. Andreev, M. Azarkin, I. Dremin, M. Kirakosyan, A. Leonidov, G. Mesyats, S. V. Rusakov, A. Baskakov, A. Belyaev, E. Boos, M. Dubinin, L. Dudko, A. Ershov, A. Gribushin, V. Klyukhin, O. Kodolova, I. Lokhtin, I. Miagkov, S. Obraztsov, S. Petrushanko, V. Savrin, A. Snigirev, I. Azhgirey, I. Bayshev, S. Bitioukov, V. Kachanov, A. Kalinin, D. Konstantinov, V. Krychkine, V. Petrov, R. Ryutin, A. Sobol, L. Tourtchanovitch, S. Troshin, N. Tyurin, A. Uzunian, A. Volkov, P. Adzic, P. Cirkovic, D. Devetak, J. Milosevic, V. Rekovic, J. Alcaraz Maestre, E. Calvo, M. Cerrada, M. Chamizo Llatas, N. Colino, B. De La Cruz, A. Delgado Peris, A. Escalante Del Valle, C. Fernandez Bedoya, J. P. Fernández Ramos, J. Flix, M. C. Fouz, P. Garcia-Abia, O. Gonzalez Lopez, S. Goy Lopez, J. M. Hernandez, M. I. Josa, E. Navarro De Martino, A. Pérez-Calero Yzquierdo, J. Puerta Pelayo, A. Quintario Olmeda, I. Redondo, L. Romero, M. S. Soares, J. F. de Trocóniz, M. Missiroli, D. Moran, J. Cuevas, J. Fernandez Menendez, S. Folgueras, I. Gonzalez Caballero, E. Palencia Cortezon, J. M. Vizan Garcia, I. J. Cabrillo, A. Calderon, J. R. Castiñeiras De Saa, E. Curras, M. Fernandez, J. Garcia-Ferrero, G. Gomez, A. Lopez Virto, J. Marco, R. Marco, C. Martinez Rivero, F. Matorras, J. Piedra Gomez, T. Rodrigo, A. Y. Rodríguez-Marrero, A. Ruiz-Jimeno, L. Scodellaro, N. Trevisani, I. Vila, R. Vilar Cortabitarte, D. Abbaneo, E. Auffray, G. Auzinger, M. Bachtis, P. Baillon, A. H. Ball, D. Barney, A. Benaglia, L. Benhabib, G. M. Berruti, P. Bloch, A. Bocci, A. Bonato, C. Botta, H. Breuker, T. Camporesi, R. Castello, M. Cepeda, G. Cerminara, M. D’Alfonso, D. d’Enterria, A. Dabrowski, V. Daponte, A. David, M. De Gruttola, F. De Guio, A. De Roeck, E. Di Marco, M. Dobson, M. Dordevic, B. Dorney, T. du Pree, D. Duggan, M. Dünser, N. Dupont, A. Elliott-Peisert, S. Fartoukh, G. Franzoni, J. Fulcher, W. Funk, D. Gigi, K. Gill, M. Girone, F. Glege, R. Guida, S. Gundacker, M. Guthoff, J. Hammer, P. Harris, J. Hegeman, V. Innocente, P. Janot, H. Kirschenmann, V. Knünz, M. J. Kortelainen, K. Kousouris, P. Lecoq, C. Lourenço, M. T. Lucchini, N. Magini, L. Malgeri, M. Mannelli, A. Martelli, L. Masetti, F. Meijers, S. Mersi, E. Meschi, F. Moortgat, S. Morovic, M. Mulders, H. Neugebauer, S. Orfanelli, L. Orsini, L. Pape, E. Perez, M. Peruzzi, A. Petrilli, G. Petrucciani, A. Pfeiffer, M. Pierini, D. Piparo, A. Racz, T. Reis, G. Rolandi, M. Rovere, M. Ruan, H. Sakulin, J. B. Sauvan, C. Schäfer, C. Schwick, M. Seidel, A. Sharma, P. Silva, M. Simon, P. Sphicas, J. Steggemann, M. Stoye, Y. Takahashi, D. Treille, A. Triossi, A. Tsirou, V. Veckalns, G. I. Veres, N. Wardle, H. K. Wöhri, A. Zagozdzinska, W. D. Zeuner, W. Bertl, K. Deiters, W. Erdmann, R. Horisberger, Q. Ingram, H. C. Kaestli, D. Kotlinski, U. Langenegger, T. Rohe, F. Bachmair, L. Bäni, L. Bianchini, B. Casal, G. Dissertori, M. Dittmar, M. Donegà, P. Eller, C. Grab, C. Heidegger, D. Hits, J. Hoss, G. Kasieczka, P. Lecomte, W. Lustermann, B. Mangano, M. Marionneau, P. Martinez Ruiz del Arbol, M. Masciovecchio, M. T. Meinhard, D. Meister, F. Micheli, P. Musella, F. Nessi-Tedaldi, F. Pandolfi, J. Pata, F. Pauss, G. Perrin, L. Perrozzi, M. Quittnat, M. Rossini, M. Schönenberger, A. Starodumov, M. Takahashi, V. R. Tavolaro, K. Theofilatos, R. Wallny, T. K. Aarrestad, C. Amsler, L. Caminada, M. F. Canelli, V. Chiochia, A. De Cosa, C. Galloni, A. Hinzmann, T. Hreus, B. Kilminster, C. Lange, J. Ngadiuba, D. Pinna, G. Rauco, P. Robmann, D. Salerno, Y. Yang, K. H. Chen, T. H. Doan, Sh. Jain, R. Khurana, M. Konyushikhin, C. M. Kuo, W. Lin, Y. J. Lu, A. Pozdnyakov, S. S. Yu, Arun Kumar, P. Chang, Y. H. Chang, Y. W. Chang, Y. Chao, K. F. Chen, P. H. Chen, C. Dietz, F. Fiori, W.-S. Hou, Y. Hsiung, Y. F. Liu, R.-S. Lu, M. Miñano Moya, J. F. Tsai, Y. M. Tzeng, B. Asavapibhop, K. Kovitanggoon, G. Singh, N. Sri manobhas, N. Suwonjandee, A. Adiguzel, S. Cerci, S. Damarseckin, Z. S. Demiroglu, C. Dozen, I. Dumanoglu, E. Eskut, S. Girgis, G. Gokbulut, Y. Guler, E. Gurpinar, I. Hos, E. E. Kangal, A. Kayis Topaksu, G. Onengut, K. Ozdemir, S. Ozturk, A. Polatoz, C. Zorbilmez, B. Bilin, S. Bilmis, B. Isildak, G. Karapinar, M. Yalvac, M. Zeyrek, E. Gülmez, M. Kaya, O. Kaya, E. A. Yetkin, T. Yetkin, A. Cakir, K. Cankocak, S. Sen, B. Grynyov, L. Levchuk, P. Sorokin, R. Aggleton, F. Ball, L. Beck, J. J. Brooke, D. Burns, E. Clement, D. Cussans, H. Flacher, J. Goldstein, M. Grimes, G. P. Heath, H. F. Heath, J. Jacob, L. Kreczko, C. Lucas, Z. Meng, D. M. Newbold, S. Paramesvaran, A. Poll, T. Sakuma, S. Seif El Nasr-storey, S. Senkin, D. Smith, V. J. Smith, K. W. Bell, A. Belyaev, C. Brew, R. M. Brown, L. Calligaris, D. Cieri, D. J. A. Cockerill, J. A. Coughlan, K. Harder, S. Harper, E. Olaiya, D. Petyt, C. H. Shepherd-Themistocleous, A. Thea, I. R. Tomalin, T. Williams, S. D. Worm, M. Baber, R. Bainbridge, O. Buchmuller, A. Bundock, D. Burton, S. Casasso, M. Citron, D. Colling, L. Corpe, P. Dauncey, G. Davies, A. De Wit, M. Della Negra, P. Dunne, A. Elwood, D. Futyan, Y. Haddad, G. Hall, G. Iles, R. Lane, R. Lucas, L. Lyons, A.-M. Magnan, S. Malik, L. Mastrolorenzo, J. Nash, A. Nikitenko, J. Pela, B. Penning, M. Pesaresi, D. M. Raymond, A. Richards, A. Rose, C. Seez, A. Tapper, K. Uchida, M. Vazquez Acosta, T. Virdee, S. C. Zenz, J. E. Cole, P. R. Hobson, A. Khan, P. Kyberd, D. Leslie, I. D. Reid, P. Symonds, L. Teodorescu, M. Turner, A. Borzou, K. Call, J. Dittmann, K. Hatakeyama, H. Liu, N. Pastika, O. Charaf, S. I. Cooper, C. Henderson, P. Rumerio, D. Arcaro, A. Avetisyan, T. Bose, D. Gastler, D. Rankin, C. Richardson, J. Rohlf, L. Sulak, D. Zou, J. Alimena, G. Benelli, E. Berry, D. Cutts, A. Ferapontov, A. Garabedian, J. Hakala, U. Heintz, O. Jesus, E. Laird, G. Landsberg, Z. Mao, M. Narain, S. Piperov, S. Sagir, R. Syarif, R. Breedon, G. Breto, M. Calderon De La Barca Sanchez, S. Chauhan, M. Chertok, J. Conway, R. Conway, P. T. Cox, R. Erbacher, C. Flores, G. Funk, M. Gardner, W. Ko, R. Lander, C. Mclean, M. Mulhearn, D. Pellett, J. Pilot, F. Ricci-Tam, S. Shalhout, J. Smith, M. Squires, D. Stolp, M. Tripathi, S. Wilbur, R. Yohay, R. Cousins, P. Everaerts, A. Florent, J. Hauser, M. Ignatenko, D. Saltzberg, E. Takasugi, V. Valuev, M. Weber, K. Burt, R. Clare, J. Ellison, J. W. Gary, G. Hanson, J. Heilman, P. Jandir, E. Kennedy, F. Lacroix, O. R. Long, M. Malberti, M. Olmedo Negrete, M. I. Paneva, A. Shrinivas, H. Wei, S. Wimpenny, B. R. Yates, J. G. Branson, G. B. Cerati, S. Cittolin, R. T. D’Agnolo, M. Derdzinski, R. Gerosa, A. Holzner, R. Kelley, D. Klein, J. Letts, I. Macneill, D. Olivito, S. Padhi, M. Pieri, M. Sani, V. Sharma, S. Simon, M. Tadel, A. Vartak, S. Wasserbaech, C. Welke, J. Wood, F. Würthwein, A. Yagil, G. Zevi Della Porta, J. Bradmiller-Feld, C. Campagnari, A. Dishaw, V. Dutta, K. Flowers, M. Franco Sevilla, P. Geffert, C. George, F. Golf, L. Gouskos, J. Gran, J. Incandela, N. Mccoll, S. D. Mullin, J. Richman, D. Stuart, I. Suarez, C. West, J. Yoo, D. Anderson, A. Apresyan, J. Bendavid, A. Bornheim, J. Bunn, Y. Chen, J. Duarte, A. Mott, H. B. Newman, C. Pena, M. Spiropulu, J. R. Vlimant, S. Xie, R. Y. Zhu, M. B. Andrews, V. Azzolini, A. Calamba, B. Carlson, T. Ferguson, M. Paulini, J. Russ, M. Sun, H. Vogel, I. Vorobiev, J. P. Cumalat, W. T. Ford, F. Jensen, A. Johnson, M. Krohn, T. Mulholland, K. Stenson, S. R. Wagner, J. Alexander, A. Chatterjee, J. Chaves, J. Chu, S. Dittmer, N. Eggert, N. Mirman, G. Nicolas Kaufman, J. R. Patterson, A. Rinkevicius, A. Ryd, L. Skinnari, L. Soffi, W. Sun, S. M. Tan, W. D. Teo, J. Thom, J. Thompson, J. Tucker, Y. Weng, P. Wittich, S. Abdullin, M. Albrow, G. Apollinari, S. Banerjee, L. A. T. Bauerdick, A. Beretvas, J. Berryhill, P. C. Bhat, G. Bolla, K. Burkett, J. N. Butler, H. W. K. Cheung, F. Chlebana, S. Cihangir, M. Cremonesi, V. D. Elvira, I. Fisk, J. Freeman, E. Gottschalk, L. Gray, D. Green, S. Grünendahl, O. Gutsche, D. Hare, R. M. Harris, S. Hasegawa, J. Hirschauer, Z. Hu, B. Jayatilaka, S. Jindariani, M. Johnson, U. Joshi, B. Klima, B. Kreis, S. Lammel, J. Lewis, J. Linacre, D. Lincoln, R. Lipton, T. Liu, R. Lopes De Sá, J. Lykken, K. Maeshima, J. M. Marraffino, S. Maruyama, D. Mason, P. McBride, P. Merkel, S. Mrenna, S. Nahn, C. Newman-Holmes, V. O’Dell, K. Pedro, O. Prokofyev, G. Rakness, E. Sexton-Kennedy, A. Soha, W. J. Spalding, L. Spiegel, S. Stoynev, N. Strobbe, L. Taylor, S. Tkaczyk, N. V. Tran, L. Uplegger, E. W. Vaandering, C. Vernieri, M. Verzocchi, R. Vidal, M. Wang, H. A. Weber, A. Whitbeck, D. Acosta, P. Avery, P. Bortignon, D. Bourilkov, A. Brinkerhoff, A. Carnes, M. Carver, D. Curry, S. Das, R. D. Field, I. K. Furic, J. Konigsberg, A. Korytov, K. Kotov, P. Ma, K. Matchev, H. Mei, P. Milenovic, G. Mitselmakher, D. Rank, R. Rossin, L. Shchutska, D. Sperka, N. Terentyev, L. Thomas, J. Wang, S. Wang, J. Yelton, S. Linn, P. Markowitz, G. Martinez, J. L. Rodriguez, A. Ackert, J. R. Adams, T. Adams, A. Askew, S. Bein, J. Bochenek, B. Diamond, J. Haas, S. Hagopian, V. Hagopian, K. F. Johnson, A. Khatiwada, H. Prosper, A. Santra, M. Weinberg, M. M. Baarmand, V. Bhopatkar, S. Colafranceschi, M. Hohlmann, H. Kalakhety, D. Noonan, T. Roy, F. Yumiceva, M. R. Adams, L. Apanasevich, D. Berry, R. R. Betts, I. Bucinskaite, R. Cavanaugh, O. Evdokimov, L. Gauthier, C. E. Gerber, D. J. Hofman, P. Kurt, C. O’Brien, I. D. Sandoval Gonzalez, P. Turner, N. Varelas, Z. Wu, M. Zakaria, J. Zhang, B. Bilki, W. Clarida, K. Dilsiz, S. Durgut, R. P. Gandrajula, M. Haytmyradov, V. Khristenko, J.-P. Merlo, H. Mermerkaya, A. Mestvirishvili, A. Moeller, J. Nachtman, H. Ogul, Y. Onel, F. Ozok, A. Penzo, C. Snyder, E. Tiras, J. Wetzel, K. Yi, I. Anderson, B. Blumenfeld, A. Cocoros, N. Eminizer, D. Fehling, L. Feng, A. V. Gritsan, P. Maksimovic, M. Osherson, J. Roskes, U. Sarica, M. Swartz, M. Xiao, Y. Xin, C. You, P. Baringer, A. Bean, C. Bruner, J. Castle, R. P. Kenny III, A. Kropivnitskaya, D. Majumder, M. Malek, W. Mcbrayer, M. Murray, S. Sanders, R. Stringer, Q. Wang, A. Ivanov, K. Kaadze, S. Khalil, M. Makouski, Y. Maravin, A. Mohammadi, L. K. Saini, N. Skhirtladze, S. Toda, D. Lange, F. Rebassoo, D. Wright, C. Anelli, A. Baden, O. Baron, A. Belloni, B. Calvert, S. C. Eno, C. Ferraioli, J. A. Gomez, N. J. Hadley, S. Jabeen, R. G. Kellogg, T. Kolberg, J. Kunkle, Y. Lu, A. C. Mignerey, Y. H. Shin, A. Skuja, M. B. Tonjes, S. C. Tonwar, A. Apyan, R. Barbieri, A. Baty, R. Bi, K. Bierwagen, S. Brandt, W. Busza, I. A. Cali, Z. Demiragli, L. Di Matteo, G. Gomez Ceballos, M. Goncharov, D. Gulhan, D. Hsu, Y. Iiyama, G. M. Innocenti, M. Klute, D. Kovalskyi, K. Krajczar, Y. S. Lai, Y.-J. Lee, A. Levin, P. D. Luckey, A. C. Marini, C. Mcginn, C. Mironov, S. Narayanan, X. Niu, C. Paus, C. Roland, G. Roland, J. Salfeld-Nebgen, G. S. F. Stephans, K. Sumorok, K. Tatar, M. Varma, D. Velicanu, J. Veverka, J. Wang, T. W. Wang, B. Wyslouch, M. Yang, V. Zhukova, A. C. Benvenuti, B. Dahmes, A. Evans, A. Finkel, A. Gude, P. Hansen, S. Kalafut, S. C. Kao, K. Klapoetke, Y. Kubota, Z. Lesko, J. Mans, S. Nourbakhsh, N. Ruckstuhl, R. Rusack, N. Tambe, J. Turkewitz, J. G. Acosta, S. Oliveros, E. Avdeeva, R. Bartek, K. Bloom, S. Bose, D. R. Claes, A. Dominguez, C. Fangmeier, R. Gonzalez Suarez, R. Kamalieddin, D. Knowlton, I. Kravchenko, F. Meier, J. Monroy, F. Ratnikov, J. E. Siado, G. R. Snow, B. Stieger, M. Alyari, J. Dolen, J. George, A. Godshalk, C. Harrington, I. Iashvili, J. Kaisen, A. Kharchilava, A. Kumar, A. Parker, S. Rappoccio, B. Roozbahani, G. Alverson, E. Barberis, D. Baumgartel, M. Chasco, A. Hortiangtham, A. Massironi, D. M. Morse, D. Nash, T. Orimoto, R. Teixeira De Lima, D. Trocino, R.-J. Wang, D. Wood, J. Zhang, S. Bhattacharya, K. A. Hahn, A. Kubik, J. F. Low, N. Mucia, N. Odell, B. Pollack, M. H. Schmitt, K. Sung, M. Trovato, M. Velasco, N. Dev, M. Hildreth, C. Jessop, D. J. Karmgard, N. Kellams, K. Lannon, N. Marinelli, F. Meng, C. Mueller, Y. Musienko, M. Planer, A. Reinsvold, R. Ruchti, N. Rupprecht, G. Smith, S. Taroni, N. Valls, M. Wayne, M. Wolf, A. Woodard, L. Antonelli, J. Brinson, B. Bylsma, L. S. Durkin, S. Flowers, A. Hart, C. Hill, R. Hughes, W. Ji, B. Liu, W. Luo, D. Puigh, M. Rodenburg, B. L. Winer, H. W. Wulsin, O. Driga, P. Elmer, J. Hardenbrook, P. Hebda, S. A. Koay, P. Lujan, D. Marlow, T. Medvedeva, M. Mooney, J. Olsen, C. Palmer, P. Piroué, D. Stickland, C. Tully, A. Zuranski, S. Malik, A. Barker, V. E. Barnes, D. Benedetti, L. Gutay, M. K. Jha, M. Jones, A. W. Jung, K. Jung, D. H. Miller, N. Neumeister, B. C. Radburn-Smith, X. Shi, J. Sun, A. Svyatkovskiy, F. Wang, W. Xie, L. Xu, N. Parashar, J. Stupak, A. Adair, B. Akgun, Z. Chen, K. M. Ecklund, F. J. M. Geurts, M. Guilbaud, W. Li, B. Michlin, M. Northup, B. P. Padley, R. Redjimi, J. Roberts, J. Rorie, Z. Tu, J. Zabel, B. Betchart, A. Bodek, P. de Barbaro, R. Demina, Y. t. Duh, Y. Eshaq, T. Ferbel, M. Galanti, A. Garcia-Bellido, J. Han, O. Hindrichs, A. Khukhunaishvili, K. H. Lo, P. Tan, M. Verzetti, J. P. Chou, E. Contreras-Campana, Y. Gershtein, T. A. Gómez Espinosa, E. Halkiadakis, M. Heindl, D. Hidas, E. Hughes, S. Kaplan, R. Kunnawalkam Elayavalli, S. Kyriacou, A. Lath, K. Nash, H. Saka, S. Salur, S. Schnetzer, D. Sheffield, S. Somalwar, R. Stone, S. Thomas, P. Thomassen, M. Walker, M. Foerster, J. Heideman, G. Riley, K. Rose, S. Spanier, K. Thapa, O. Bouhali, A. Castaneda Hernandez, A. Celik, M. Dalchenko, M. De Mattia, A. Delgado, S. Dildick, R. Eusebi, J. Gilmore, T. Huang, T. Kamon, V. Krutelyov, R. Mueller, I. Osipenkov, Y. Pakhotin, R. Patel, A. Perloff, L. Perniè, D. Rathjens, A. Rose, A. Safonov, A. Tatarinov, K. A. Ulmer, N. Akchurin, C. Cowden, J. Damgov, C. Dragoiu, P. R. Dudero, J. Faulkner, S. Kunori, K. Lamichhane, S. W. Lee, T. Libeiro, S. Undleeb, I. Volobouev, Z. Wang, E. Appelt, A. G. Delannoy, S. Greene, A. Gurrola, R. Janjam, W. Johns, C. Maguire, Y. Mao, A. Melo, H. Ni, P. Sheldon, S. Tuo, J. Velkovska, Q. Xu, M. W. Arenton, P. Barria, B. Cox, B. Francis, J. Goodell, R. Hirosky, A. Ledovskoy, H. Li, C. Neu, T. Sinthuprasith, X. Sun, Y. Wang, E. Wolfe, F. Xia, C. Clarke, R. Harr, P. E. Karchin, C. Kottachchi Kankanamge Don, P. Lamichhane, J. Sturdy, D. A. Belknap, D. Carlsmith, S. Dasu, L. Dodd, S. Duric, B. Gomber, M. Grothe, M. Herndon, A. Hervé, P. Klabbers, A. Lanaro, A. Levine, K. Long, R. Loveless, A. Mohapatra, I. Ojalvo, T. Perry, G. A. Pierro, G. Polese, T. Ruggles, T. Sarangi, A. Savin, A. Sharma, N. Smith, W. H. Smith, D. Taylor, P. Verwilligen, N. Woods, T. Aziz, S. Banerjee, S. Bhowmik, R. M. Chatterjee, R. K. Dewanjee, S. Dugad, S. Ganguly, S. Ghosh, M. Guchait, A. Gurtu, Sa. Jain, G. Kole, S. Kumar, B. Mahakud, M. Maity, G. Majumder, K. Mazumdar, S. Mitra, G. B. Mohanty, B. Parida, T. Sarkar, N. Sur, B. Sutar, N. Wickramage, CMS Collaboration

**Affiliations:** 1 Yerevan Physics Institute, Yerevan, Armenia; 2 Institut für Hochenergiephysik, Vienna, Austria; 3 National Centre for Particle and High Energy Physics, Minsk, Belarus; 4 Universiteit Antwerpen, Antwerp, Belgium; 5 Vrije Universiteit Brussel, Brussels, Belgium; 6 Université Libre de Bruxelles, Brussels, Belgium; 7 Ghent University, Ghent, Belgium; 8 Université Catholique de Louvain, Louvain-la-Neuve, Belgium; 9 Université de Mons, Mons, Belgium; 10 Centro Brasileiro de Pesquisas Fisicas, Rio de Janeiro, Brazil; 11 Universidade do Estado do Rio de Janeiro, Rio de Janeiro, Brazil; 12 Universidade Estadual Paulista, Universidade Federal do ABC, São Paulo, Brazil; 13 Institute for Nuclear Research and Nuclear Energy, Sofia, Bulgaria; 14 University of Sofia, Sofia, Bulgaria; 15 Beihang University, Beijing, China; 16 Institute of High Energy Physics, Beijing, China; 17 State Key Laboratory of Nuclear Physics and Technology, Peking University, Beijing, China; 18 Universidad de Los Andes, Bogotá, Colombia; 19 Faculty of Electrical Engineering, Mechanical Engineering and Naval Architecture, University of Split, Split, Croatia; 20 Faculty of Science, University of Split, Split, Croatia; 21 Institute Rudjer Boskovic, Zagreb, Croatia; 22 University of Cyprus, Nicosia, Cyprus; 23 Charles University, Prague, Czech Republic; 24 Universidad San Francisco de Quito, Quito, Ecuador; 25 Egyptian Network of High Energy Physics, Academy of Scientific Research and Technology of the Arab Republic of Egypt, Cairo, Egypt; 26 National Institute of Chemical Physics and Biophysics, Tallinn, Estonia; 27 Department of Physics, University of Helsinki, Helsinki, Finland; 28 Helsinki Institute of Physics, Helsinki, Finland; 29 Lappeenranta University of Technology, Lappeenranta, Finland; 30 IRFU, CEA, Université Paris-Saclay, Gif-sur-Yvette, France; 31 Laboratoire Leprince-Ringuet, Ecole Polytechnique, IN2P3-CNRS, Palaiseau, France; 32 Institut Pluridisciplinaire Hubert Curien, Université de Strasbourg, Université de Haute Alsace Mulhouse, CNRS/IN2P3, Strasbourg, France; 33 Centre de Calcul de l’Institut National de Physique Nucleaire et de Physique des Particules, CNRS/IN2P3, Villeurbanne, France; 34 Institut de Physique Nucléaire de Lyon, Université de Lyon, Université Claude Bernard Lyon 1, CNRS-IN2P3, Villeurbanne, France; 35 Georgian Technical University, Tbilisi, Georgia; 36 Tbilisi State University, Tbilisi, Georgia; 37 I. Physikalisches Institut, RWTH Aachen University, Aachen, Germany; 38 III. Physikalisches Institut A, RWTH Aachen University, Aachen, Germany; 39 III. Physikalisches Institut B, RWTH Aachen University, Aachen, Germany; 40 Deutsches Elektronen-Synchrotron, Hamburg, Germany; 41 University of Hamburg, Hamburg, Germany; 42 Institut für Experimentelle Kernphysik, Karlsruhe, Germany; 43 Institute of Nuclear and Particle Physics (INPP), NCSR Demokritos, Aghia Paraskevi, Greece; 44 National and Kapodistrian University of Athens, Athens, Greece; 45 University of Ioánnina, Ioannina, Greece; 46 MTA-ELTE Lendület CMS Particle and Nuclear Physics Group, Eötvös Loránd University, Budapest, Hungary; 47 Wigner Research Centre for Physics, Budapest, Hungary; 48 Institute of Nuclear Research ATOMKI, Debrecen, Hungary; 49 Institute of Physics, University of Debrecen, Debrecen, Hungary; 50 National Institute of Science Education and Research, Bhubaneswar, India; 51 Panjab University, Chandigarh, India; 52 University of Delhi, Delhi, India; 53 Saha Institute of Nuclear Physics, Kolkata, India; 54 Bhabha Atomic Research Centre, Mumbai, India; 55 Indian Institute of Science Education and Research (IISER), Pune, India; 56 Institute for Research in Fundamental Sciences (IPM), Tehran, Iran; 57 University College Dublin, Dublin, Ireland; 58 INFN Sezione di Bari, Università di Bari, Politecnico di Bari, Bari, Italy; 59 INFN Sezione di Bologna, Università di Bologna, Bologna, Italy; 60 INFN Sezione di Catania, Università di Catania, Catania, Italy; 61 INFN Sezione di Firenze, Università di Firenze, Florence, Italy; 62 INFN Laboratori Nazionali di Frascati, Frascati, Italy; 63 INFN Sezione di Genova, Università di Genova, Genoa, Italy; 64 INFN Sezione di Milano-Bicocca, Università di Milano-Bicocca, Milan, Italy; 65 INFN Sezione di Napoli, Università di Napoli ‘Federico II’ , Napoli, Italy, Università della Basilicata , Potenza, Italy, Università G. Marconi, Rome, Italy; 66 INFN Sezione di Padova, Università di Padova, Padua, Italy, Università di Trento, Trento, Italy; 67 INFN Sezione di Pavia, Università di Pavia, Pavia, Italy; 68 INFN Sezione di Perugia, Università di Perugia, Perugia, Italy; 69 INFN Sezione di Pisa, Università di Pisa, Scuola Normale Superiore di Pisa, Pisa, Italy; 70 INFN Sezione di Roma, Università di Roma, Rome, Italy; 71 INFN Sezione di Torino, Università di Torino, Turin, Italy, Università del Piemonte Orientale, Novara, Italy; 72 INFN Sezione di Trieste, Università di Trieste, Trieste, Italy; 73 Kangwon National University, Chunchon, Korea; 74 Kyungpook National University, Daegu, Korea; 75 Chonbuk National University, Jeonju, Korea; 76 Institute for Universe and Elementary Particles, Chonnam National University, Kwangju, Korea; 77 Korea University, Seoul, Korea; 78 Seoul National University, Seoul, Korea; 79 University of Seoul, Seoul, Korea; 80 Sungkyunkwan University, Suwon, Korea; 81 Vilnius University, Vilnius, Lithuania; 82 National Centre for Particle Physics, Universiti Malaya, Kuala Lumpur, Malaysia; 83 Centro de Investigacion y de Estudios Avanzados del IPN, Mexico City, Mexico; 84 Universidad Iberoamericana, Mexico City, Mexico; 85 Benemerita Universidad Autonoma de Puebla, Puebla, Mexico; 86 Universidad Autónoma de San Luis Potosí, San Luis Potosí, Mexico; 87 University of Auckland, Auckland, New Zealand; 88 University of Canterbury, Christchurch, New Zealand; 89 National Centre for Physics, Quaid-I-Azam University, Islamabad, Pakistan; 90 National Centre for Nuclear Research, Swierk, Poland; 91 Faculty of Physics, Institute of Experimental Physics, University of Warsaw, Warsaw, Poland; 92 Laboratório de Instrumentação e Física Experimental de Partículas, Lisbon, Portugal; 93 Joint Institute for Nuclear Research, Dubna, Russia; 94 Petersburg Nuclear Physics Institute, Gatchina, St. Petersburg, Russia; 95 Institute for Nuclear Research, Moscow, Russia; 96 Institute for Theoretical and Experimental Physics, Moscow, Russia; 97 National Research Nuclear University ‘Moscow Engineering Physics Institute’ (MEPhI), Moscow, Russia; 98 P.N. Lebedev Physical Institute, Moscow, Russia; 99 Skobeltsyn Institute of Nuclear Physics, Lomonosov Moscow State University, Moscow, Russia; 100 State Research Center of Russian Federation, Institute for High Energy Physics, Protvino, Russia; 101 Faculty of Physics and Vinca Institute of Nuclear Sciences, University of Belgrade, Belgrade, Serbia; 102 Centro de Investigaciones Energéticas Medioambientales y Tecnológicas (CIEMAT), Madrid, Spain; 103 Universidad Autónoma de Madrid, Madrid, Spain; 104 Universidad de Oviedo, Oviedo, Spain; 105 Instituto de Física de Cantabria (IFCA), CSIC-Universidad de Cantabria, Santander, Spain; 106 CERN, European Organization for Nuclear Research, Geneva, Switzerland; 107 Paul Scherrer Institut, Villigen, Switzerland; 108 Institute for Particle Physics, ETH Zurich, Zurich, Switzerland; 109 Universität Zürich, Zurich, Switzerland; 110 National Central University, Chung-Li, Taiwan; 111 National Taiwan University (NTU), Taipei, Taiwan; 112 Department of Physics, Faculty of Science, Chulalongkorn University, Bangkok, Thailand; 113 Physics Department, Science and Art Faculty, Cukurova University, Adana, Turkey; 114 Physics Department, Middle East Technical University, Ankara, Turkey; 115 Bogazici University, Istanbul, Turkey; 116 Istanbul Technical University, Istanbul, Turkey; 117 Institute for Scintillation Materials, National Academy of Science of Ukraine, Kharkov, Ukraine; 118 National Scientific Center, Kharkov Institute of Physics and Technology, Kharkov, Ukraine; 119 University of Bristol, Bristol, UK; 120 Rutherford Appleton Laboratory, Didcot, UK; 121 Imperial College, London, UK; 122 Brunel University, Uxbridge, UK; 123 Baylor University, Waco, USA; 124 The University of Alabama, Tuscaloosa, USA; 125 Boston University, Boston, USA; 126 Brown University, Providence, USA; 127 University of California, Davis, Davis, USA; 128 University of California, Los Angeles, USA; 129 University of California, Riverside, Riverside, USA; 130 University of California, San Diego, La Jolla, USA; 131 Department of Physics, University of California, Santa Barbara, Santa Barbara, USA; 132 California Institute of Technology, Pasadena, USA; 133 Carnegie Mellon University, Pittsburgh, USA; 134 University of Colorado Boulder, Boulder, USA; 135 Cornell University, Ithaca, USA; 136 Fermi National Accelerator Laboratory, Batavia, USA; 137 University of Florida, Gainesville, USA; 138 Florida International University, Miami, USA; 139 Florida State University, Tallahassee, USA; 140 Florida Institute of Technology, Melbourne, USA; 141 University of Illinois at Chicago (UIC), Chicago, USA; 142 The University of Iowa, Iowa City, USA; 143 Johns Hopkins University, Baltimore, USA; 144 The University of Kansas, Lawrence, USA; 145 Kansas State University, Manhattan, USA; 146 Lawrence Livermore National Laboratory, Livermore, USA; 147 University of Maryland, College Park, USA; 148 Massachusetts Institute of Technology, Cambridge, USA; 149 University of Minnesota, Minneapolis, USA; 150 University of Mississippi, Oxford, USA; 151 University of Nebraska-Lincoln, Lincoln, USA; 152 State University of New York at Buffalo, Buffalo, USA; 153 Northeastern University, Boston, USA; 154 Northwestern University, Evanston, USA; 155 University of Notre Dame, Notre Dame, USA; 156 The Ohio State University, Columbus, USA; 157 Princeton University, Princeton, USA; 158 University of Puerto Rico, Mayaguez, USA; 159 Purdue University, West Lafayette, USA; 160 Purdue University Calumet, Hammond, USA; 161 Rice University, Houston, USA; 162 University of Rochester, Rochester, USA; 163 Rutgers, The State University of New Jersey, Piscataway, USA; 164 University of Tennessee, Knoxville, USA; 165 Texas A&M University, College Station, USA; 166 Texas Tech University, Lubbock, USA; 167 Vanderbilt University, Nashville, USA; 168 University of Virginia, Charlottesville, USA; 169 Wayne State University, Detroit, USA; 170 University of Wisconsin-Madison, Madison, WI USA; 171 Tata Institute of Fundamental Research, Mumbai, India; 172 CERN, 1211 Geneva 23, Switzerland

## Abstract

Measurements of the associated production of a $\mathrm{Z}$ boson with at least one jet originating from a b quark in proton–proton collisions at $\sqrt{s} = 8\,\text {TeV}$ are presented. Differential cross sections are measured with data collected by the CMS experiment corresponding to an integrated luminosity of 19.8$\,\text {fb}^{-1}$. $\mathrm{Z}$ bosons are reconstructed through their decays to electrons and muons. Cross sections are measured as a function of observables characterizing the kinematics of the $\mathrm{b}$ jet and the $\mathrm{Z}$ boson. Ratios of differential cross sections for the associated production with at least one $\mathrm{b}$ jet to the associated production with any jet are also presented. The production of a $\mathrm{Z}$ boson with at least two $\mathrm{b}$ jets is investigated, and differential cross sections are measured for the dijet system. Results are compared to theoretical predictions, testing two different flavour schemes for the choice of initial-state partons.

## Introduction

The associated production of vector bosons and jets (V+jets) in hadronic collisions is a large background source in measurements of several standard model (SM) processes, Higgs boson studies, and many searches for physics beyond the SM. Its description constitutes an important benchmark for perturbative quantum chromodynamics (pQCD) predictions. Differential cross sections as a function of kinematic observables characterizing V+jets topologies are sensitive to the contributions from both the hard scattering process and the associated soft QCD radiation, as well as to the parton distribution functions (PDFs). Among the V+jets processes, the case in which a $\mathrm{Z}/\gamma ^{*}$ boson is produced in association with $\mathrm{b}$ quarks, $\mathrm {p}\mathrm {p}\rightarrow \mathrm{Z} +({\ge }1\mathrm{b})$, hereafter denoted as $\mathrm{Z} (1\mathrm{b})$, is particularly interesting. Antiquarks are also assumed in the notation, and the $\mathrm{Z}/\gamma ^{*}$ interference contribution is considered to be part of the process. Within the SM, the $\mathrm{Z} (1\mathrm{b})$ final state is the dominant background for studies of the associated production of Higgs and $\mathrm{Z}$ bosons, in which the Higgs boson decays into a $\mathrm{b} \overline{\mathrm{b}}$ pair [1]. Many physics scenarios beyond the SM predict final states with $\mathrm{b}$ quarks and $\mathrm{Z}$ bosons: new generations of heavy quarks ($\mathrm{b} ^{\prime }, \mathrm{t} ^{\prime }$) decaying into $\mathrm{Z} (1\mathrm{b})$ [2], supersymmetric Higgs bosons produced in association with $\mathrm{b}$ quarks [3], and extended SM scenarios with additional SU(2) doublets with enhanced $\mathrm{Z} \mathrm{b} \overline{\mathrm{b}}$ coupling [4]. The study of the associated production of $\mathrm{Z}$ bosons and $\mathrm{b}$ quark jets may also provide information useful in describing an analogous process where a $\mathrm {W}$ boson is produced, which is more difficult to measure because of higher background contamination.

This paper presents measurements of associated production of a $\mathrm{Z}$ boson and b quark jets using proton–proton collision data at 8$\,\text {TeV}$ collected with the CMS detector, corresponding to an integrated luminosity of 19.8$\,\text {fb}^{-1}$. The $\mathrm{Z}$ boson is reconstructed through its leptonic decay into an electron or muon pair, while the presence of $\mathrm{b}$ quarks is inferred from the characteristics of jets (denoted as $\mathrm{b}$ jets) that originate from their hadronization products and subsequent decays. In order to characterize $\mathrm{Z} (1\mathrm{b})$ production, fiducial differential cross sections are measured as a function of five kinematic observables: the transverse momentum $p_{\mathrm {T}}$ and pseudorapidity $\eta$ of the highest-$p_{\mathrm {T}}$ $\mathrm{b}$ jet, the $\mathrm{Z}$ boson $p_{\mathrm {T}}$, the scalar sum of the transverse momenta of all jets regardless of the flavour of the parton producing them ($H_{\mathrm {T}}$), and the azimuthal angular difference between the direction of the $\mathrm{Z}$ boson and the highest-$p_{\mathrm {T}}$ $\mathrm{b}$ jet ($\varDelta \phi _{\mathrm{Z} \mathrm{b}}$). Ratios of the differential cross sections for $\mathrm{Z} (1\mathrm{b})$ and $\mathrm{Z}$+jets production, inclusive in jet flavour, are also measured as a function of these five observables. The cancellation of several systematic uncertainties in the cross section ratio allows an even more precise comparison with theory than the differential cross sections themselves.

Events with at least two $\mathrm{b}$ jets, henceforth $\mathrm{Z} (2\mathrm{b})$, contribute as background sources to other SM and beyond-SM processes. The production dynamics of this kind of event are studied through the measurement of the fiducial differential cross section as a function of observables characterizing the kinematic properties of the dijet system formed by the leading and subleading (in $p_{\mathrm {T}}$) $\mathrm{b}$ jets: the $p_{\mathrm {T}}$ of these two jets; the $\mathrm{Z}$ boson $p_{\mathrm {T}}$; the invariant masses of the $\mathrm{b} \mathrm{b}$ and $\mathrm{Z} \mathrm{b} \mathrm{b}$ systems ($M_{{\mathrm{b} \mathrm{b}}}$ and $M_{\mathrm{Z} \mathrm{b} \mathrm{b}}$ respectively); the angle $\varDelta \phi _{{\mathrm{b} \mathrm{b}}}$ between the two $\mathrm{b}$ jets in the plane transverse to the beam axis and their separation in the $\eta$–$\phi$ plane ($\varDelta R_{{\mathrm{b} \mathrm{b}}}$); the distance in the $\eta$–$\phi$ plane between the $\mathrm{Z}$ boson and the closer $\mathrm{b}$ jet ($\varDelta R^{\text {min}}_{\mathrm{Z} \mathrm{b}}$); and the asymmetry in the distances in the $\eta$–$\phi$ plane between the $\mathrm{Z}$ boson and the closer versus farther $\mathrm{b}$ jets ($A_{\mathrm{Z} \mathrm{b} \mathrm{b}}$).

Previously, the cross section for the associated production of $\mathrm{Z}$ bosons and $\mathrm{b}$ jets was measured in proton–antiproton collisions by the CDF [5] and D0 [6] Collaborations at the Fermilab Tevatron and in proton–proton collisions at a centre-of-mass energy of 7$\,\text {TeV}$ by the ATLAS [7] and CMS [8] Collaborations at the CERN LHC. The CMS Collaboration also studied $\mathrm{Z} (2\mathrm{b})$ production by explicitly reconstructing $\mathrm{b}$ hadron decays [9], in order to explore the region where $\mathrm{b}$ quarks are emitted in an almost collinear topology. Previous measurements of the ratio of the $\mathrm{Z} (1\mathrm{b})$ to the $\mathrm{Z}$+jets inclusive cross section were published by the D0 Collaboration [10].

The paper is organized as follows: Sect. 2 is dedicated to the description of the CMS apparatus and Sect. 3 to the data and simulated samples used in the analysis. Section 4 discusses the lepton, jet, and $\mathrm{b}$ jet reconstruction and the event selection. Section 5 discusses background estimation, while Sect. 6 is dedicated to the description of the unfolding procedure to correct data for detector effects. Section 7 presents a discussion of the systematic uncertainties. In Sect. 8 the measured differential cross sections and the corresponding ratios are presented, together with a discussion of the comparison with theoretical predictions. Finally, the results are summarized in Sect. 9.

## The CMS detector

A detailed description of the CMS detector, together with the definition of the coordinate system used and the relevant kinematic variables, can be found in Ref. [11]. The central feature of the CMS apparatus is a superconducting solenoid of 6 $\text {m}$ internal diameter. The field volume houses a silicon tracker, a crystal electromagnetic calorimeter (ECAL), and a brass and scintillator hadron calorimeter(HCAL), each composed of a barrel and two endcap sections. The magnet flux-return yoke is instrumented with muon detectors. The silicon tracker measures charged particles within the pseudorapidity range $|\eta | < 2.5$. It consists of 1440 silicon pixel and 15 148 silicon strip detector modules and is located in the 3.8 $\text {T}$ field of the superconducting solenoid. For nonisolated particles of $1< p_{\mathrm {T}} < 10~\text {GeV}$ and $|\eta | < 1.4$, the track resolutions are typically 1.5% in $p_{\mathrm {T}}$ and 25–90 (45–150)$~\upmu \text {m}$ in the transverse (longitudinal) impact parameter [12]. The electron momentum is estimated by combining the energy measurement in the ECAL with the momentum measurement in the tracker. The momentum resolution for electrons with $p_{\mathrm {T}} \approx 45~\text {GeV}$ from $\mathrm{Z} \rightarrow \mathrm {e}\mathrm {e}$ decays ranges from 1.7% for nonshowering electrons in the barrel region to 4.5% for showering electrons in the endcaps [13]. Muons are measured in the pseudorapidity range $|\eta | < 2.4$, with detection planes made using three technologies: drift tubes, cathode strip chambers, and resistive plate chambers. Matching muons to tracks measured in the silicon tracker results in a relative transverse momentum resolution for muons with $20<p_{\mathrm {T}} < 100~\text {GeV}$ of 1.3–2.0% in the barrel and better than 6% in the endcaps. The $p_{\mathrm {T}}$ resolution in the barrel is better than 10% for muons with $p_{\mathrm {T}}$ up to 1$\,\text {TeV}$ [14]. Forward calorimeters extend the pseudorapidity coverage provided by the barrel and endcap detectors.

The CMS detector uses a two-level trigger system. The first level of the system, composed of custom hardware processors, uses information from the calorimeters and muon detectors to select the most interesting events in a fixed time interval of less than 4 $\,\upmu \text {s}$. The high-level trigger processor farm further decreases the event rate from around 100 $\text {kHz}$ to less than 1 $\text {kHz}$ before data storage.

## Event simulation

The associated production of a $\mathrm{Z}$ boson and jets is experimentally reconstructed as two opposite-sign same-flavour electrons or muons accompanied by jets and can be mimicked by various background sources: $\mathrm{t}\overline{\mathrm{t}}$ events, dibosons ($\mathrm {W}\mathrm {W}$, $\mathrm {W}\mathrm{Z}$, $\mathrm{Z} \mathrm{Z}$) and $\mathrm {W}$ bosons produced in association with jets, single top quark events, as well as $\mathrm{Z}$+jets events in which the $\mathrm{Z}$ boson decays into $\mathrm {\tau }^+\mathrm {\tau }^-$. Diboson events with a leptonic $\mathrm{Z}$ boson decay and jets produced in the hadronic decay of the other vector boson are not considered as part of the signal. Samples of simulated events are used to model both the signal and the background processes. The MadGraph 5.1.3.30 [15] event generator is used to simulate $\mathrm{Z}$+jets (including jets from $\mathrm{b}$ quarks), $\mathrm {W}$+jets, and $\mathrm{t}\overline{\mathrm{t}}$ events; this generator implements a leading-order (LO) matrix element calculation with up to four (three) additional partons in the final state for V+jets ($\mathrm{t}\overline{\mathrm{t}}$) events, using the CTEQ6L1 PDF set [16], which is based on the five flavour scheme (5FS). A detailed discussion is given in Sect. 8.2. The parton-level events are interfaced with pythia version 6.424 [17] for parton showering, hadronization, and description of the multiple-parton interactions (MPIs). The pythia6 Z2* tune, which is based on the CTEQ6L1 PDF set, is used [18]. The matrix element and parton shower calculations are matched using the $k_{\mathrm {t}}$-MLM algorithm [19]. The cross section inclusive in jet multiplicity is rescaled to its next-to-next-to-leading-order (NNLO) prediction, computed with fewz 3.1 [20, 21] for the $\mathrm{Z}$+jets and $\mathrm {W}$+jets processes, and with the calculation of Ref. [22] for the $\mathrm{t}\overline{\mathrm{t}}$ process. To study systematic uncertainties, signal events are also generated using MadGraph5_aMC@NLO [23] version 2.2.1, with next-to-leading-order (NLO) matrix elements for zero, one, and two additional partons merged with the FxFx algorithm [24], interfaced with pythia version 8.205 [25] for showering and hadronization. In this case the NNPDF 3.0 NLO PDF set [26] is used. Depending on the flavours included in the matrix element calculation of the event or produced in the parton shower through gluon splitting, the inclusive $\mathrm{Z}$+jets sample can be divided into $\mathrm{Z}$+b quark, c quark, and light-flavour (u, d, s quark and gluon) jet subsamples. As explained in Sect. 6, the jet flavour identification is based on the particle content of the final state.

Diboson events are simulated with pythia6, and the inclusive cross section rescaled to the NLO prediction provided by mcfm [27]. The single top quark contribution is evaluated using powheg-box version 1.0 [28–32] interfaced with pythia6 for parton showering, hadronization, and MPI description. The contribution of multijet events is evaluated using pythia6 generated events and found to be negligible.

Generated events are processed with a simulation of the CMS detector based on the Geant4 toolkit [33]. Signals induced by additional $\mathrm {p}\mathrm {p}$ interactions in the same or adjacent bunch crossings, referred to as pileup, are simulated using events generated with pythia6. The pileup distribution in simulation is adjusted in order to reproduce the collision rates observed in data. During the 2012 data taking, the average pileup rate was about 21 interactions per bunch crossing.

## Event selection

The analysis is based on an online trigger selection requiring events to contain a pair of electron or muon candidates with asymmetric minimum thresholds on their transverse momenta. These threshold settings depended on the instantaneous luminosity and reached maximum values of 17$~\text {GeV}$ for the leading lepton and 8$~\text {GeV}$ for the subleading one. Events are required to contain a $\mathrm{Z}$ boson, reconstructed through its decay into an electron or muon pair, produced in association with at least one or at least two hadronic jets. For the $\mathrm{Z} (1\mathrm{b})$ and $\mathrm{Z} (2\mathrm{b})$ event selections the jets are also required to be identified as originating from the hadronization of a $\mathrm{b}$ quark.

All the measured particles are reconstructed using the particle-flow (PF) algorithm [34, 35]. The particle-flow event algorithm reconstructs and identifies each individual particle with an optimized combination of information from the various elements of the CMS detector. The energy of photons is obtained directly from the ECAL measurement, corrected for zero-suppression effects. The energy of electrons is evaluated from a combination of the electron momentum at the primary interaction vertex as determined by the tracker, the energy of the corresponding ECAL cluster, and the energy sum of all bremsstrahlung photons spatially compatible with originating from the electron track. The transverse momentum of the muons is obtained from the curvature of the corresponding track. The energy of charged hadrons is determined from a combination of the momentum measured in the tracker and the matching ECAL and HCAL energy deposits, corrected for zero-suppression effects and for the response functions of the calorimeters to hadronic showers. Finally, the energy of neutral hadrons is obtained from the corresponding corrected ECAL and HCAL energies.

The reconstructed leptons selected as candidate decay products of the $\mathrm{Z}$ boson must match those that triggered the event and must be associated with the primary vertex of the event, defined as the reconstructed vertex with the largest sum of $p_{\mathrm {T}} ^2$ of its constituent tracks. Reconstructed electrons must satisfy a set of selection criteria designed to minimize misidentification at a desired efficiency level [13]; the discriminating observables include the measured shower shape in the ECAL and the spatial matching between the electromagnetic deposit in the calorimeter and the reconstructed track associated with it. Additional requirements on electron tracks are used to reject products of photon conversions. Electron isolation criteria exploit the full PF-based event reconstruction, using particles within a cone around the electron direction with radius $\varDelta R= \sqrt{\smash [b]{(\varDelta \phi )^2 + (\varDelta \eta )^2}} = 0.3$. The isolation requirement is defined by $I_{\text{ rel }}=(I_{\text{ charged }} + I_{\text{ photon }}+I_{\text{ neutral }})/p_{\mathrm {T}} ^{\mathrm {e}} < 0.15$, where $I_{\text{ charged }}$ is the scalar $p_{\mathrm {T}}$ sum of all the charged hadrons, $I_{\text{ photon }}$ is the scalar $p_{\mathrm {T}}$ sum of all the photons, and $I_{\text{ neutral }}$ the scalar sum of $p_{\mathrm {T}}$ of all the neutral hadrons in the cone of interest. The notation $p_{\mathrm {T}} ^{\mathrm {e}}$ refers to the transverse momentum of the reconstructed electron. Pileup can add extra particles, which affect the isolation variable. Accordingly, only charged particles originating from the reconstructed primary vertex are used in the calculation of $I_{\text{ charged }}$. The photon and neutral hadronic contribution to the isolation variable coming from pileup is subtracted using the jet area approach [36]. Electrons must have $p_{\mathrm {T}} ^{\mathrm {e}} > 20~\text {GeV}$ and be reconstructed within the pseudorapidity range $|\eta |<1.44$ and $1.57<|\eta |<2.4$, which exclude the barrel-endcap transition region.

Muon identification criteria are based on the fit quality for tracks measured in the tracker and the muon detector [14]. Further selection criteria are added in order to reject muons from cosmic rays. Muon isolation is computed using all particles reconstructed by the PF algorithm within a cone of radius $\varDelta R= 0.4$ around the muon direction, requiring $I_{\text{ rel }}=(I_{\text{ charged }}+I_{\text{ photon }}+I_{\text{ neutral }})/p_{\mathrm {T}} ^{\mathrm {\mu }} < 0.2$. Muons must have $p_{\mathrm {T}} ^{\mathrm {\mu }} > 20~\text {GeV}$ and $|\eta |<2.4$. As in the case of electrons, charged particles not originating from the primary vertex are excluded from the isolation calculation. The pileup contribution to $I_{\text{ photon }}$ and $I_{\text {neutral}}$ is estimated as half of the corresponding charged hadronic component and is subtracted in the definition of the $I_{\text{ rel }}$ variable.

The efficiencies for lepton trigger, reconstruction, identification, and isolation are measured with the “tag-and-probe” technique [37] as a function of the lepton $\eta$ and $p_{\mathrm {T}}$. A sample of events containing a $\mathrm{Z}$ boson decaying into $\mathrm {e}^+\mathrm {e}^-$ or $\mu ^+\mu ^-$ is used for these studies. Efficiency corrections (“scale factors”) of up to 1.2% (7.3%), dependent on lepton $p_{\mathrm {T}}$ and $\eta$, are applied to account for differences in the estimated efficiencies between data and simulation in the electron (muon) channel.

The pair of selected same-flavour, opposite-sign, highest-$p_{\mathrm {T}}$ isolated leptons is retained as a $\mathrm{Z}$ boson candidate if the invariant mass $M_{\ell \ell }$ of the pair lies within the 71–111$~\text {GeV}$ mass interval. The overall efficiency of the trigger and event selection within the fiducial acceptance is 88% for dimuons and 58% for dielectrons.

Jets are reconstructed using the anti-$k_{\mathrm {t}}$ algorithm [38, 39] with a distance parameter of 0.5. In order to suppress the contribution from pileup interactions, charged particles not associated with the primary vertex are excluded from the clustering. Jets are required to be in the tracking acceptance region $|\eta |<2.4$ and to have $p_{\mathrm {T}} > 30~\text {GeV}$, thereby reducing the contribution from the underlying event to less than 5%, where jets have a softer $p_{\mathrm {T}}$ spectrum compared to jets from the hard scattering process. Jets with a distance $\varDelta R< 0.5$ from the closer lepton used for the $\mathrm{Z}$ boson decay reconstruction are not considered in the analysis. The jet energy scale (JES) is calibrated using a factorized approach as described in Refs. [40, 41]. The jet energy resolution (JER) in data is known to be worse than in the simulation; therefore the simulated resolution is degraded to compensate for this effect as a function of the jet kinematics [40, 41].

Jets from $\mathrm{b}$ quarks are identified using the combined secondary vertex (CSV) b tagging algorithm [42], a multivariate classifier that makes use of information about reconstructed secondary vertices as well as the impact parameters of the associated tracks with respect to the primary vertex to discriminate $\mathrm{b}$ jets from $\mathrm{c}$ and light-flavour jets. The threshold applied to the discriminating variable gives a b tagging efficiency of about 50% and a misidentification probability of 0.1% for light jets and 1% for $\mathrm{c}$ jets. Scale factors, measured in multijet events and dependent on jet $p_{\mathrm {T}}$, are used to correct the b, c, and light-flavour jet efficiencies in the simulation to match those observed in the data [42]. The scale factors for $\mathrm{b}$ jets are determined using samples of events enriched in such a flavour of jets. This enrichment is obtained including both multijet events containing a muon geometrically associated with a jet, with high probability of originating from the semileptonic decay of a $\mathrm{b}$ hadron, and leptonic and semileptonic $\mathrm{t}\overline{\mathrm{t}}$ events, where the leading $p_{\mathrm {T}}$ jets are usually $\mathrm{b}$ jets. The scale factors are around 0.93, slowly decreasing for jets with $p_{\mathrm {T}}$ above 120$~\text {GeV}$. The scale factors for $\mathrm{c}$ jets are assumed the same as for $\mathrm{b}$ jets, with an uncertainty twice as large. Relatively pure samples of $\mathrm{c}$ jets from $\mathrm {W}+\mathrm{c}$ events, selected using identified muons within the jet, are used to validate this assumption. For light-flavour jets, the same CSV algorithm yields scale factors between 1.1 and 1.4, depending on the jet $p_{\mathrm {T}}$. The calculation is based on tracks with negative signed impact parameter and secondary vertices with negative signed decay lengths, where the sign is defined by the relative direction of the jet and the particle momentum. Finally, events are selected if they contain a $\mathrm{Z}$ boson candidate and at least one $\mathrm{b}$-tagged jet.

The missing transverse momentum vector ${\vec {p}}_{\mathrm {T}}^{\text {miss}}$ is defined as the projection on the plane perpendicular to the beams of the negative vector sum of the momenta of all reconstructed particles in an event. Its magnitude is referred to as $E_{\mathrm {T}}^{\text {miss}}$. The $E_{\mathrm {T}}^{\text {miss}}$ significance, introduced in Refs. [43, 44], offers an event-by-event assessment of the consistency of the observed missing energy with zero, given the reconstructed content of the event and known measurement resolutions. In order to suppress the background contamination from $\mathrm{t}\overline{\mathrm{t}}$ production, events with $E_{\mathrm {T}}^{\text {miss}}$ significance greater than 30 are vetoed. This requirement provides a 13% $\mathrm{t}\overline{\mathrm{t}}$ background rejection with no loss in signal efficiency.

**Fig. 1:**
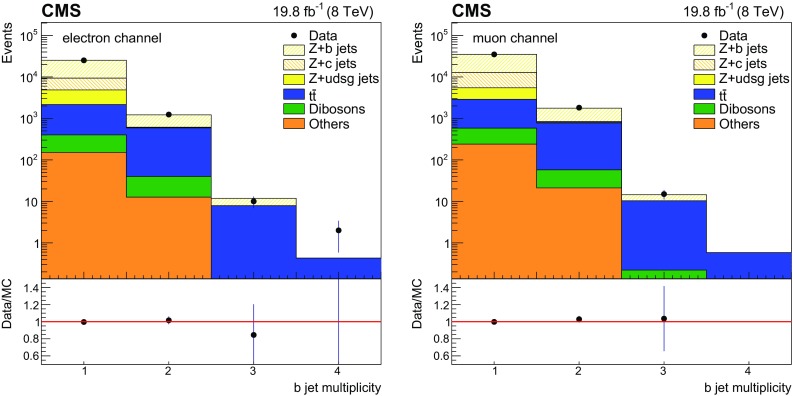
Exclusive $\mathrm{b}$-tagged jet multiplicity distributions for $\mathrm{Z} (1\mathrm{b})$ events, for the electron (*left*) and muon (*right*) decay channel of $\mathrm{Z}$ boson. *Error bars* account for statistical uncertainties in data in the *upper plots* and in both data and simulation in the *bottom ratio plots*, that show the data to MC ratio

**Fig. 2:**
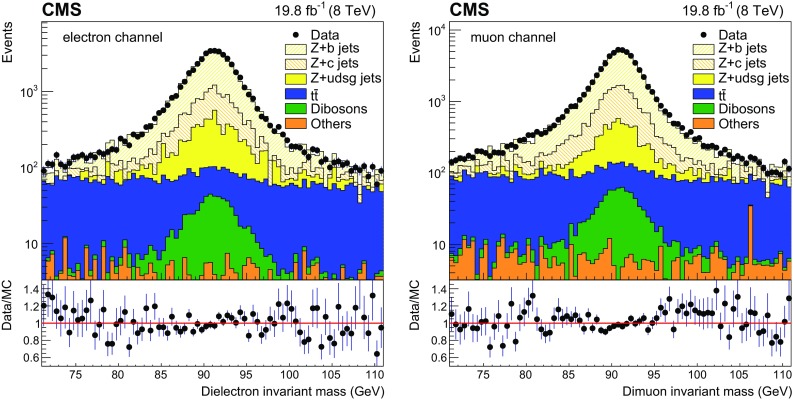
Dilepton invariant mass distributions for $\mathrm{Z} (1\mathrm{b})$ events, for the electron (*left*) and muon (*right*) $\mathrm{Z}$ boson decay channels. *Error bars* account for statistical uncertainties in data in the *upper plots* and in both data and simulation in the *bottom ratio plots*, that show the data to MC ratio

The $\mathrm{Z} (1\mathrm{b})$ event selection described above yields 26443 (36843) events for the dielectron (dimuon) channels. The exclusive $\mathrm{b}$-tagged jet multiplicity and invariant mass distributions of the same flavour dilepton are presented in Figs. 1 and 2, for the $\mathrm{Z} (1\mathrm{b})$ event selection for electron and muon respectively. Data are compared with the simulations where the $\mathrm{Z}$+jets events are described by MadGraph+pythia6, and good agreement is observed. In all figures, the simulated events are reweighted by scale factors in order to compensate for the residual data-to-simulation discrepancies in lepton selection efficiency, JES and JER calibration, and b tagging efficiency. The background contributions from $\mathrm{Z}$+jets and $\mathrm{t}\overline{\mathrm{t}}$ events as adjusted in Sect. 5 are included in Figs. 1 and 2.

## Background estimation

A Drell–Yan event in which a $\mathrm{Z}$ boson decays into $\tau ^+\tau ^-$ may contribute to the dielectron or dimuon signal events if both $\mathrm {\tau }$ leptons decay into electrons or muons. These events are treated as a background source and, being at the few per mil level, their contribution is evaluated from simulation.

The process $\mathrm {p}\mathrm {p}\rightarrow \mathrm{t}\overline{\mathrm{t}} \rightarrow \mathrm {W}^+ \mathrm{b} \mathrm {W}^-{\overline{\mathrm{b}}}\rightarrow \ell ^+\ell ^-\mathrm{b} {\overline{\mathrm{b}}}+E_{\mathrm {T}}^{\text {miss}}$ is the dominant non-Drell–Yan background source. The $\mathrm{t}\overline{\mathrm{t}}$ background contribution is estimated separately for $\mathrm{Z}$+jets, $\mathrm{Z} (1\mathrm{b})$, and $\mathrm{Z} (2\mathrm{b})$ events by using the signal selection criteria to produce samples of $\mathrm {e}\mathrm {\mu }$ pairs, which are enriched in $\mathrm{t}\overline{\mathrm{t}}$ events with negligible signal contamination. For each measured observable these samples provide the estimates of the $\mathrm{t}\overline{\mathrm{t}}$ background; residual non-$\mathrm{t}\overline{\mathrm{t}}$ backgrounds in them, amounting to about 29, 8 and 2% respectively, are subtracted using the simulated prediction. The integrals of such estimates need to be rescaled by the ratio of the same-flavour lepton to $\mathrm {e}\mathrm {\mu }$ yields. This ratio is determined using control samples for both the same-flavour lepton and $\mathrm {e}\mathrm {\mu }$ selections by inverting the $E_{\mathrm {T}}^{\text {miss}}$ significance requirement, namely, $E_{\mathrm {T}}^{\text {miss}}$ significance >30. For the same-flavour lepton samples, this selection removes the contribution from the signal processes, while enhancing the fraction of $\mathrm{t}\overline{\mathrm{t}}$ events in the sample. The residual contamination from other non-$\mathrm{t}\overline{\mathrm{t}}$ processes is similar in the same-lepton and $\mathrm {e}\mathrm {\mu }$ selections, amounting to about 20, 7, 3% respectively, and is again taken into account using the simulation. The ratio of the $\mathrm {e}\mathrm {\mu }$ to the $\mathrm {e}\mathrm {e}$ or $\mathrm {\mu }\mathrm {\mu }$ yields in the control samples is used to rescale the estimates of this background source for each lepton channel separately. The ratio is determined as the scaling factor for the normalization of the binned dilepton invariant mass ($M_{\ell \ell }$) distribution in the $\mathrm {e}\mathrm {\mu }$ sample that minimizes the difference of this distribution from the corresponding same-lepton-flavour $M_{\ell \ell }$ distribution in a least-square fit procedure. The fit of the $M_{\ell \ell }$ distribution is performed in the sideband regions 50–84$~\text {GeV}$ and 100–200$~\text {GeV}$, to avoid any assumption about the $M_{\ell \ell }$ shape for both different and same-flavour lepton pairs in the $\mathrm{Z}$ peak region.

The remaining background sources are estimated using simulation. Diboson events may mimic the $\mathrm{Z}$+b final state when one or more leptons are not reconstructed or when a $\mathrm {W}$ or $\mathrm{Z}$ boson decays hadronically into a $\mathrm{q}\overline{\mathrm{q}}$ pair (in particular a $\mathrm{Z}$ boson may decay into a genuine $\mathrm{b} \overline{\mathrm{b}}$ pair). Single top quarks produced in association with either a $\mathrm {W}$ boson or one or more $\mathrm{b}$ jets may also generate a signal-like signature. These events, together with $\mathrm {W}$+jets, can mimic the signal if a lepton of the same flavour is produced in the hadronization or if a hadron is misidentified. The contribution of multijet events is found to be negligible, as has been previously observed in other similar $\mathrm{Z}$+jets analyses [45].

After subtraction of all non-Drell–Yan background contributions, the extraction of the $\mathrm{Z} (1\mathrm{b})$ and $\mathrm{Z} (2\mathrm{b})$ event yields requires an evaluation of the purity of the b tagging selection, i.e. the fraction of selected Drell–Yan events in which the desired number of $\mathrm{b}$-tagged jets, at least one or at least two, originate from the hadronization of a corresponding number of $\mathrm{b}$ quarks. This fraction is determined from a study of the secondary vertex mass distribution of the leading $\mathrm{b}$-tagged jet, defined as the invariant mass of all the charged particles associated with its secondary vertices, assuming the pion mass for each considered particle. This evaluation is done separately for dielectron and dimuon final states to avoid correlations between channels and to simplify the combination. The secondary vertex mass distributions for $\mathrm{b}$, $\mathrm{c}$, and light-flavour jets produced in association with $\mathrm{Z}$ bosons are obtained from the simulation based on the MadGraph event generator interfaced with pythia6 by using the 5FS scheme for PDFs. The sum of the distributions is fitted to the observed distribution with an extended binned likelihood, after subtraction of all non-Drell–Yan background contributions, by varying the three normalization scale factors $c_{\mathrm {b}}$, $c_{\mathrm {c}}$, $c_{\mathrm {udsg}}$ for the various components. The $c_{\mathrm {c}}$, $c_{\mathrm {udsg}}$ factors are used for the subtraction of the respective components. This procedure reduces the dependence on the normalization of the $\mathrm{b}$ hadron production and decay in the simulation because the expected shape of the secondary vertex mass distribution is used. In the case of the $\mathrm{Z} (2\mathrm{b})$ selection, as it can be seen in Fig. 1, the contamination from c and light-flavour jets is negligible and is subtracted using simulation; only the $c_{{\mathrm{b} \mathrm{b}}}$ scaling factor for the genuine double $\mathrm{b}$ jet component is determined from the fit, and it is used only to correct the relative proportion of $\mathrm{Z} (1\mathrm{b})$ and $\mathrm{Z} (2\mathrm{b})$ events in the simulation, as discussed in Sect. 6.

**Fig. 3:**
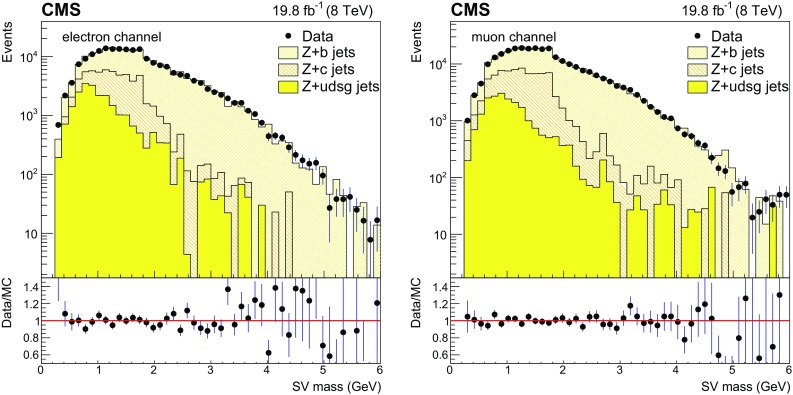
Distributions of the secondary vertex (SV) mass of the leading jet after the $\mathrm{Z} (1\mathrm{b})$ selection with the $\mathrm{Z}$ boson decaying into electrons (*left*) and muons (*right*). The subsamples corresponding to $\mathrm{b}$-tagged jets originating from $\mathrm{b}$, c, and light-flavour quarks or gluons are shown, with normalizations determined in the fit to data. Non-Drell–Yan background sources are subtracted. *Error bars* account for statistical uncertainties in data in the *upper plots* and in both data and simulation in the *bottom ratio plots*

The results of the fit to the secondary vertex mass distributions are presented in Fig. 3 for the $\mathrm{Z} (1\mathrm{b})$ analysis, showing the flavour composition in each channel. Data-to-simulation scale factors, as determined by the fit, are given in Table 1 for both event selections and $\mathrm{Z}$ boson decay channels. The flavour composition of the selected sample after the scale factor corrections for the $\mathrm{Z} (1\mathrm{b})$ samples is also shown.

The $\mathrm{b}$-flavour contribution is constrained by the high secondary vertex mass region of the distribution of the CSV discriminating variable, while the $\mathrm{c}$-flavour contribution is mostly important in the region between 1 and 2$~\text {GeV}$, and the light-flavour contribution below 1$~\text {GeV}$. This results in a strong anticorrelation both between the $\mathrm{b}$- and $\mathrm{c}$-flavour and between $\mathrm{c}$- and light-flavour contributions, with an estimated correlation coefficient of about −0.6 in both cases, whereas the correlation between the $\mathrm{b}$- and light-flavour contributions is negligible. As a consequence, a fluctuation in the small $\mathrm{c}$ quark component may cause a difference in the scale factors between different lepton channels.

**Table 1:** Normalization scale factors and post-fit fractions for b, c and light-flavour (u, d, s quark and gluon) components in the selected $\mathrm{Z} (1\mathrm{b})$ events, and scale factor for b in the selected $\mathrm{Z} (2\mathrm{b})$ events, obtained from a fit to the secondary vertex mass distribution for dielectron and dimuon final states. The quoted uncertainties are statistical only

Event selection	$c_{\mathrm {b}}$	$c_{\mathrm {c}}$	$c_{\mathrm {udsg}}$	$\mathrm{Z} (1\mathrm{b})$ (%)	Z+c (%)	Z+udsg (%)
$\mathrm{Z} (1\mathrm{b})$ ($\mathrm {e}\mathrm {e}$)	$0.91\pm 0.02$	$1.29\pm 0.13$	$1.70\pm 0.21$	$69.5\pm 1.8$	$19.0\pm 2.0$	$11.4\pm 1.4$
$\mathrm{Z} (1\mathrm{b})$ ($\mathrm {\mu }\mathrm {\mu }$)	$0.91\pm 0.02$	$1.51\pm 0.12$	$1.18\pm 0.19$	$69.7\pm 1.5$	$22.4\pm 1.8$	$7.9\pm 1.2$

Event selection	$c_{{\mathrm{b} \mathrm{b}}}$					
$\mathrm{Z} (2\mathrm{b})$ ($\mathrm {e}\mathrm {e}$)	$1.18\pm 0.12$					
$\mathrm{Z} (2\mathrm{b})$ ($\mathrm {\mu }\mathrm {\mu }$)	$1.17\pm 0.09$					

The signal yield for $\mathrm{Z} (1\mathrm{b})$ events is therefore obtained, for each bin of a distribution, from the selected event yield $N^{\text {selected}}$ as

$$\begin{aligned} N_{{\mathrm{Z}} (1{\mathrm{b}})}= & {} N^{\text{selected}}_{\mathrm{Z} (1\mathrm{b})}- N_{\mathrm{t}\overline{\mathrm{t}}} - N_{\text {Dibosons}}^{\mathrm {MC}} - N_{\text {Others}}^{\mathrm {MC}} \\ &-\, c_{\mathrm {c}} N_{{\mathrm{Z}}+{\mathrm{c}}}^{\mathrm {MC}} - c_{\mathrm {udsg}} N_{{\mathrm{Z}}+{\mathrm{udsg}}}^{\mathrm {MC}}, \end{aligned}$$

where $N_{\mathrm{t}\overline{\mathrm{t}}}$, $N_{\text {Dibosons}}^{\mathrm {MC}}$, and $N_{\text {Others}}^{\mathrm {MC}}$ are the $\mathrm{t}\overline{\mathrm{t}}$, diboson, and other background contributions respectively, $c_{\mathrm {c}} N_{\mathrm {Z+c}}^{\mathrm {MC}}$ and $c_{\mathrm {udsg}} N_{\mathrm{Z}+\mathrm{udsg}}^\mathrm{MC}$ are the numbers of Drell–Yan events in which the b-tagged jets originate from either a c or a light-flavour parton, and the scale factors multiply the event yields predicted by the simulation. For the calculation of the $\mathrm{Z} (2\mathrm{b})$ event yield a similar procedure is applied:

$$\begin{aligned} {N_{\mathrm{Z} (2\mathrm{b})} = N^{\text {selected}}_{\mathrm{Z} (2\mathrm{b})} - N_{\mathrm{t}\overline{\mathrm{t}}} - N_{\text {Dibosons}}^{\mathrm {MC}} - N_{\text {Others}}^{\mathrm {MC}} .} \end{aligned}$$

The $c_\mathrm {c}$ and $c_\mathrm {udsg}$ scale factors are also re-evaluated from subsamples obtained by dividing the ranges of the studied observables into wide intervals, in order to study a possible correlation with the observables themselves. The statistical uncertainty of these scale factors depends on the chosen observable and binning, ranging from a factor of 2 up to 10 relative to the size of the uncertainty of the default values obtained with the full sample. Because no statistically significant dependence is observed, the scale factors estimated from the overall sample are used.

The amount of background in the final event selection, estimated with the procedures discussed above, can be observed in Fig. 1. For the $\mathrm{Z} (1\mathrm{b})$ selection, in the electrons (muons) samples the Z+$\mathrm{c}$ contribution amounts to about 17% (20%), the Z+light flavour jets (including gluons) to 10% (7%), and the $\mathrm{t}\overline{\mathrm{t}}$ to 9% (8%). Other background contributions are globally below the 2% level. The $\mathrm{Z} (1\mathrm{b})$ contribution in the corresponding selected sample is about 62% (63%) for the electrons (muons) channel.

## Unfolding

The differential event yields are corrected for event selection efficiencies and for detector resolution effects back to the stable-particle level. For this purpose, the singular value decomposition (SVD) [46] unfolding technique, implemented in the RooUnfold toolkit [47], is used. The unfolding procedure is based on a response matrix, which describes the relationship between the particle levels and measured values of a given observable due to the detector resolution and acceptance. The response matrix is calculated using $\mathrm{Z} (1\mathrm{b})$ events that are generated with MadGraph in the 5FS, interfaced to pythia6, and followed by the detector simulation. Response matrices are computed separately for the $\mathrm{Z} (1\mathrm{b})$ and $\mathrm{Z} (2\mathrm{b})$ selections. The proportion of events with exactly one or at least two b quarks in the simulation is reweighted to match that observed in data, as determined by the $c_{{\mathrm{b} \mathrm{b}}}$ scaling factor.

Fiducial cross sections are defined, based on event generator predictions at the particle level, for leptons and jets reconstructed from the collection of all stable final-state particles, using the same selection criteria as the data analysis. The two leptons (electrons or muons) with the highest transverse momentum and with $p_{\mathrm {T}} > 20~\text {GeV}$ and $|\eta | < 2.4$ are selected as $\mathrm{Z}$ boson decay products if their invariant mass is in the range of 71–111$~\text {GeV}$. Electromagnetic final-state radiation effects are taken into account in the generator-level lepton definition by clustering all photons in a cone of radius $\varDelta R= 0.1$ around the final-state leptons. The leptons selected as $\mathrm{Z}$ boson decay products are then removed from the particle collection used for the jet clustering at the generator level. The remaining particles, excluding neutrinos, are clustered into jets using the anti-$k_{\mathrm {t}}$ algorithm with a distance parameter of 0.5. Generated jets are selected if their distance from the leptons forming the $\mathrm{Z}$ boson candidate is larger than $\varDelta R= 0.5$. Jets originating from the hadronization of $\mathrm{b}$ quarks are selected if a $\mathrm{b}$ hadron is an ancestor of one of the particles clustered in it, and this $\mathrm{b}$ hadron has a distance from the jet in the $\eta$-$\phi$ plane of $\varDelta R\le 0.5$. Jets and $\mathrm{b}$ jets are selected if they have $p_{\mathrm {T}} > 30~\text {GeV}$ and lie in the pseudorapidity range $|\eta | < 2.4$.

As a cross-check of the SVD technique, the unfolding is also performed with the iterative D’Agostini method [48], leading to compatible results within the statistical uncertainties.

## Systematic uncertainties

Several sources of systematic uncertainty affect the cross section measurement: the JES and JER, the calculation of the unfolding response matrix, the estimation of the $\mathrm{b}$ quark fraction, the background subtraction, the event selection efficiencies, the pileup description, and the integrated luminosity. For every source other than the luminosity, the full analysis procedure is repeated after the variation of the corresponding input values, and the difference of the extracted cross section with respect to the central measurement is used as an estimate of the uncertainty due to that source. The uncertainties are symmetrized, if not already symmetric. The systematic uncertainties in the measured $\mathrm{Z} (1\mathrm{b})$ and $\mathrm{Z} (2\mathrm{b})$ differential cross sections are summarized in Table 2 and in Tables 3 and 4, respectively.

Reconstructed jet energies must be corrected for several effects, such as pileup contamination, instrumental noise, nonuniformities and nonlinearities in the detector response, and flavour composition. The resulting uncertainty depends on the transverse momentum and pseudorapidity of the jet. The systematic effect due to the application of JES corrections in the data is estimated by increasing and decreasing the correction parameters deviation from their nominal values by one standard deviation. The uncertainty for the JER correction is evaluated in the same way.

For the cross section measurement in a given bin, the systematic uncertainty induced by the model used in the unfolding procedure is evaluated as the difference between the standard result and that obtained with an alternative model for unfolding, namely MadGraph5_aMC@NLO interfaced with pythia8. This alternative model implements NLO hard scattering matrix elements, compared to the LO matrix elements of MadGraph interfaced to pythia6, and also includes different details of the underlying event, hadronization, and particle decay descriptions compared to the default choice. In order to evaluate the genuine model-induced effects, the statistical uncertainties from the two simulated samples are subtracted in quadrature from the difference; any negative results so obtained are replaced with zero. The uncertainty associated with the size of the simulated sample used to compute the response matrix elements is determined by producing replicas of the matrix whose elements are fluctuated according to a Poisson distribution.

The uncertainty induced by the secondary vertex mass fit, used to extract the true flavour composition of the $\mathrm{Z} (1\mathrm{b})$ sample, is twofold. One part is due to the statistical uncertainty in the $c_\mathrm {c}$, $c_\mathrm {udsg}$ scale factors, whose effect is estimated by varying them up and down by one standard deviation, taking into account their correlation. This source of uncertainty is considered as part of the statistical uncertainty, because it is due to the finite size of the collision data sample. The other part stems from the choice of the simulation model for the shape of the secondary vertex mass distributions. This choice affects also the correction of the relative proportion of different $\mathrm{b}$ multiplicities provided by the scale factor $c_{\mathrm{b} \mathrm{b}}$. In addition, a systematic uncertainty is associated, for both $\mathrm{Z} (1\mathrm{b})$ and $\mathrm{Z} (2\mathrm{b})$ samples, with the modelling of the $\mathrm{c}$ quark and light-flavour contributions to each measured observable. Both of these model-induced uncertainties, collectively indicated in the tables as “c, udsg background model”, are estimated by replacing the default model given by MadGraph 5FS interfaced with pythia6 with MadGraph5_aMC@NLO 5FS interfaced with pythia8. The scale factors, which are determined from the alternative model, are in statistical agreement for dielectron and dimuon channels within one standard deviation. The difference between the results obtained with the two models is therefore considered as safely accounting for possible residual discrepancies between data and simulation.

For each lepton channel the systematic uncertainties in the lepton efficiency calculations for triggering, reconstruction, identification, and isolation are estimated from the $\mathrm{Z} \rightarrow \ell \ell$ “tag-and-probe” measurements of data-to-simulation efficiency scale factors. The global effect of the systematic uncertainty related to the scale factors is 1.5% in the dielectron final state and 2% in the dimuon final state. The uncertainties in the b tagging efficiency scale factors include contributions from the pileup contamination, the gluon splitting rate in simulation ($\mathrm{g}\rightarrow \mathrm{b} \overline{\mathrm{b}}$), varied by ${\pm }50\%$, and the energy fraction carried by the b hadrons in the hadronization (varied by ${\pm }5\%$) [42]. The global value of the $\mathrm{b}$ tagging systematic uncertainty amounts to 3% per $\mathrm{b}$-tagged jet. Scale factors for $\mathrm{c}$ jets, assumed equal to those for $\mathrm{b}$ jets, are assigned an uncertainty twice as large as for the $\mathrm{b}$ jets.

The simulation is reweighted according to the generated primary vertex multiplicity and the instantaneous luminosity in data to reproduce the observed primary vertex multiplicity distribution, and provide a reliable representation of pileup. The minimum-bias event cross section in simulation is tuned to provide the best agreement between data and simulation in the vertex multiplicity distribution of $\mathrm{Z} \rightarrow \mathrm {\mu }\mathrm {\mu }$ events. The uncertainty associated with this procedure is estimated by varying this minimum-bias cross section value by 5%.

The uncertainty in the $\mathrm{t}\overline{\mathrm{t}}$ background normalization is derived from the statistical uncertainties of the same-flavour and $\mathrm {e}\mathrm {\mu }$ control samples and is included in the total statistical uncertainty. The systematic uncertainty related to the diboson background ($\mathrm{Z} \mathrm{Z}$, $\mathrm {W}\mathrm {W}$, $\mathrm {W}\mathrm{Z}$) is evaluated by varying the theoretical production cross sections by ${\pm } 15\%$ of their central values, corresponding to about three standard deviations of the overall theoretical normalization uncertainty and covering the typical differences between the theoretical and measured values. In addition, the statistical uncertainty induced by the limited size of simulation samples is taken into account.

The systematic uncertainty in the integrated luminosity is 2.6% [49].

In the ratios of $\mathrm{Z} (1\mathrm{b})$ and $\mathrm{Z} (2\mathrm{b})$ to the inclusive $\mathrm{Z}$+jets cross sections, the uncertainties are simultaneously propagated to both the numerator and denominator, taking correlations into account. The uncertainties in the energy scale, resolution, and efficiency corrections for reconstructed leptons and jets are considered to be fully correlated, as is the uncertainty in the integrated luminosity. Tables 2, 3 and 4 summarize the ranges of variation of the uncertainties for each observable measured with the $\mathrm{Z} (1\mathrm{b})$ and $\mathrm{Z} (2\mathrm{b})$ samples.

**Table 2:** Uncertainties (in percent) in the differential cross sections as a function of the leading $\mathrm{b}$ jet $p_{\mathrm {T}}$ and $|\eta |$, the $\mathrm{Z}$ boson $p_{\mathrm {T}}$, $H_{\mathrm {T}}$, and $\varDelta \phi _{\mathrm{Z} \mathrm{b}}$ between the $\mathrm{Z}$ boson and the leading $\mathrm{b}$ jet, for the $\mathrm{Z} (1\mathrm{b})$ sample

Uncertainty (%)	${\mathrm{d}\sigma }/{\mathrm{d}p_{\mathrm {T}}}$	${\mathrm{d}\sigma }/{\mathrm{d}|\eta |}$	${\mathrm{d}\sigma }/{\mathrm{d}p_{\mathrm {T}} ^{{\mathrm{Z}}}}$	${\mathrm{d}\sigma }/{\mathrm{d}H_{\mathrm {T}}}$	${\mathrm{d}\sigma }/{\mathrm{d}\varDelta \phi _{\mathrm{Z} \mathrm{b}}}$
JER	0.3–1.7	0.1–0.6	0.2–2.6	0.4–1.9	0.1–2.2
JES	0.5–4.8	0.7–5.3	0.5–7.7	0.6–5.2	0.4–4.2
Unfolding (MC model)	0.0–19.2	0.2–2.2	0.0–18.1	0.0–10.2	0.0–9.2
Unfolding (MC statistics)	1.4–10.2	1.1–2.7	1.8–8.3	1.3–7.6	1.2–6.1
c, udsg background model	0.0–6.1	0.0–7.0	0.0–19.9	0.7–7.5	0.0–10.9
Electron (muon) efficiency	1.5 (2.0)	1.5 (2.0)	1.5 (2.0)	1.5 (2.0)	1.5 (2.0)
$\mathrm{b}$ tagging efficiency	3.0	3.0	3.0	3.0	3.0
Pileup	0.2–4.3	0.6–1.4	0.4–2.0	0.2–2.3	0.2–1.6
Background (systematic)	0.1–0.4	0.1–0.3	0.1–0.6	0.2–0.3	0.1–0.3
Background (statistical)	1.2–7.2	1.0–2.5	1.5–5.8	1.3–4.6	1.2–5.9
Integrated luminosity	2.6	2.6	2.6	2.6	2.6
Total syst. uncertainty (%)	5.5–21.7	5.2–10.6	5.6–22.8	8.4–13.8	6.0–13.3
Total stat. uncertainty (%)	2.6–8.8	3.0–5.4	2.9–8.6	3.1–6.0	3.1–7.0

**Table 3:** Uncertainties (in percent) in the differential cross sections as a function of the leading and subleading $\mathrm{b}$ jet $p_{\mathrm {T}}$, the $\mathrm{Z}$ boson $p_{\mathrm {T}}$, the invariant mass of the two $\mathrm{b}$-tagged jets, and the invariant mass of the $\mathrm{Z}$ boson and the two $\mathrm{b}$-tagged jets, for the $\mathrm{Z} (2\mathrm{b})$ sample

Uncertainty (%)	${\mathrm{d}\sigma }/{\mathrm{d}p_{\mathrm {T}} ^{\text {leading}}}$	${\mathrm{d}\sigma }/{\mathrm{d}p_{\mathrm {T}} ^{\text {subleading}}}$	${\mathrm{d}\sigma }/{\mathrm{d}p_{\mathrm {T}} ^{\mathrm{Z}}}$	${\mathrm{d}\sigma }/{\mathrm{d}M_{{\mathrm{b} \mathrm{b}}}}$	${\mathrm{d}\sigma }/{\mathrm{d}M_{\mathrm{Z} \mathrm{b} \mathrm{b}}}$
JER	0.3–8.3	0.7–7.9	0.1–3.8	0.9–4.1	2.9–12.0
JES	4.4–17.0	7.7–23.3	3.1–20.3	6.7–15.3	3.8–16.2
Unfolding (MC model)	0.0–74.4	0.0–52.6	0.0–53.6	0.0–37.8	0.0–57.3
Unfolding (MC statistics)	8.0–39.4	9.0–35.8	8.8–27.0	7.6–28.0	10.0–20.8
c, udsg background model	0.0–17.3	0.0–16.1	0.0–15.5	0.0–18.5	0.0–10.2
Electron (muon) efficiency	1.5 (2.0)	1.5 (2.0)	1.5 (2.0)	1.5 (2.0)	1.5 (2.0)
$\mathrm{b}$ tagging efficiency	6.0	6.0	6.0	6.0	6.0
Pileup	0.4–14.1	0.3–11.4	1.3–9.6	1.1–5.7	0.2–4.3
Background (systematic)	0.3–0.9	0.1–0.7	0.3–1.2	0.0–1.4	0.3–1.3
Background (statistical)	3.1–17.4	4.0–24.2	4.2–15.0	4.3–15.0	5.8–10.2
Integrated luminosity	2.6	2.6	2.6	2.6	2.6
Total syst. uncertainty (%)	17.2–89.4	19.7–61.7	17.8–56.6	14.5–52.9	17.9–65.4
Total stat. uncertainty (%)	6.1–34.1	7.6–44.5	10.4–23.5	7.9–28.0	11.2–19.9

**Table 4:** Uncertainties (in percent) in the differential cross sections as a function of $\varDelta R$ and $\varDelta \phi$ between the two $\mathrm{b}$-tagged jets, $\varDelta R$ between the $\mathrm{Z}$ boson and the closer $\mathrm{b}$-tagged jet, and the asymmetry $A_{\mathrm{Z} \mathrm{b} \mathrm{b}}$, for the $\mathrm{Z} (2\mathrm{b})$ sample

Uncertainty (%)	${\mathrm{d}\sigma }/{\mathrm{d}\varDelta \phi _{\mathrm{b} \mathrm{b}}}$	${\mathrm{d}\sigma }/{\mathrm{d}\varDelta R_{{\mathrm{b} \mathrm{b}}}}$	${\mathrm{d}\sigma }/{\mathrm{d}\varDelta R^{\text {min}}_{\mathrm{Z} \mathrm{b}}}$	${\mathrm{d}\sigma }/{\mathrm{d}A_{{\mathrm{Z} \mathrm{b} \mathrm{b}}}}$
JER	0.8–2.0	1.0–5.3	0.6–6.1	0.6–4.2
JES	5.6–10.7	6.6–20.5	4.2–13.1	5.1–9.1
Unfolding (MC model)	0.0–47.0	0.0–206	0.0–50.6	2.6–33.1
Unfolding (MC statistics)	6.3–11.5	6.4–30.7	8.2–25.6	7.5–30.5
c, udsg background model	0.0–3.4	0.0–10.3	0.0–14.2	0.0–12.3
Electron (muon) efficiency	1.5 (2.0)	1.5 (2.0)	1.5 (2.0)	1.5 (2.0)
$\mathrm{b}$ tagging efficiency	6.0	6.0	6.0	6.0
Pileup	0.4–2.4	1.3–3.5	0.5–4.6	1.2–6.1
Background (systematic)	0.1–0.8	0.1–0.8	0.2–1.3	0.2–0.7
Background (statistical)	3.4–5.0	3.7–9.4	3.6–15.9	3.3–8.8
Integrated luminosity	2.6	2.6	2.6	2.6
Total syst. uncertainty (%)	13.0–50.5	12.5–209	14.2–59.5	13.6–47.2
Total stat. uncertainty (%)	6.9–10.1	7.5–17.6	7.4–33.1	6.6–18.4

## Results and comparison with theoretical predictions

### Observables

Differential cross sections as a function of a number of kinematic observables are measured in order to characterize the production mechanisms of $\mathrm{Z} (1\mathrm{b})$ events.

For $\mathrm{Z} (1\mathrm{b})$ events, five kinematic observables are studied. First, $p_{\mathrm {T}}$ and $|\eta |$ of the leading-$p_{\mathrm {T}}$ $\mathrm{b}$ jet are measured, together with the $\mathrm{Z}$ boson $p_{\mathrm {T}}$. The distributions of these variables are directly sensitive to the $\mathrm{b}$ quark PDF and initial-state gluon splitting and may show differences between different PDF flavour schemes. Searches for physics processes beyond the SM in Lorentz-boosted topology events depend on precise knowledge of the $\mathrm{Z}$ boson $p_{\mathrm {T}}$ distribution. The scalar sum $H_{\mathrm {T}}$ of the transverse momenta of all selected jets, regardless of their flavour, is related to the structure of the hadronic system recoiling against the boson. The measurement of this observable at high values is potentially sensitive to the presence of intermediate heavy particles decaying hadronically, as predicted, for example, in some SUSY scenarios. Finally, the topology of the system composed of the $\mathrm{Z}$ boson and $\mathrm{b}$ jet is studied by measuring the cross section as a function of the azimuthal angular separation between the direction of the $\mathrm{Z}$ boson and the direction of the highest-$p_{\mathrm {T}}$ $\mathrm{b}$ jet, $\varDelta \phi _{\mathrm{Z} \mathrm{b}}$. This observable is also sensitive to the presence of boosted particles decaying into a $\mathrm{Z}$ boson and $\mathrm{b}$ quarks.

Ratios of the differential cross sections for $\mathrm{Z} (1\mathrm{b})$ and $\mathrm{Z}$+jets events, inclusive in the jet flavour, are also measured:

$$\begin{aligned} R(x)=\frac{\mathrm{d}\sigma (\mathrm{Z} {+({\ge }1\mathrm{b})})/\mathrm{d}x}{\mathrm{d}\sigma (\mathrm{Z} {+}\text {jets})/\mathrm{d}x}, \end{aligned}$$

with *x* representing one of the five observables described above. The inclusive $\mathrm{Z}$+jets event selection is defined by releasing the requirement of a b-tagged jet in the $\mathrm{Z} (1\mathrm{b})$ selection. In these ratios the kinematic observables referring to the highest-$p_{\mathrm {T}}$ b-tagged jet from the $\mathrm{Z} (1\mathrm{b})$ sample are used in the numerator, while for the denominator the observables related to the highest-$p_{\mathrm {T}}$ jet from the $\mathrm{Z}$+jet sample are examined. Several systematic uncertainties cancel in the ratios, allowing a precise comparison with theory.

For $\mathrm{Z} (2\mathrm{b})$ events, the cross section is measured as a function of the transverse momenta of the $\mathrm{Z}$ boson and of the leading and subleading $\mathrm{b}$ jets. In addition, the cross section is studied as a function of several variables explicitly related to the topology of the final state consisting of a $\mathrm{Z}$ boson and the two highest-$p_{\mathrm {T}}$ $\mathrm{b}$ jets. The invariant mass ${M_{{\mathrm{b} \mathrm{b}}}}$ of the $\mathrm{b} \mathrm{b}$ system and the invariant mass $M_{\mathrm{Z} \mathrm{b} \mathrm{b}}$ of the $\mathrm{Z} \mathrm{b} \mathrm{b}$ system are studied, because their distributions are sensitive to the presence of heavy intermediate particles.

Angular correlations between the $\mathrm{b}$ jets and between each $\mathrm{b}$ jet and the $\mathrm{Z}$ boson are described by four observables, also studied in Ref. [9]. The azimuthal angular separation $\varDelta \phi _{{\mathrm{b} \mathrm{b}}}$ between the directions of the two $\mathrm{b}$ jets in the transverse plane is useful to identify back-to-back configurations of the $\mathrm{b}$ quarks. The distance between the directions of the two $\mathrm{b}$ jets in the $\eta$-$\phi$ plane is defined as $\varDelta R_{{\mathrm{b} \mathrm{b}}} = \sqrt{\smash [b]{(\varDelta \eta _{{\mathrm{b} \mathrm{b}}})^{2}+(\varDelta \phi _{{\mathrm{b} \mathrm{b}}})^{2}}}$, where $\varDelta \eta _{{\mathrm{b} \mathrm{b}}}$ is the separation in pseudorapidity between the two $\mathrm{b}$ jets. This variable is sensitive to the different production mechanisms of the $\mathrm{Z} (2\mathrm{b})$ final-state $\mathrm{b}$ quarks. In particular, it is useful to discriminate between the contributions whose scattering amplitudes are dominated by terms involving gluon splitting, $\mathrm{g}\rightarrow \mathrm{b} \overline{\mathrm{b}}$, and those where a $\mathrm{Z}$ boson is emitted from one of the final-state $\mathrm{b}$ quarks. The process $\mathrm{q} \overline{\mathrm{q}} \rightarrow \mathrm{Z} \mathrm{b} \overline{\mathrm{b}}$ contributes to both cases, while $\mathrm{q} \mathrm{g} \rightarrow \mathrm{Z} \mathrm{b} \overline{\mathrm{b}} \mathrm {X}$ (with $\mathrm {X}$ an additional parton) contributes to the former and $\mathrm{g} \mathrm{g} \rightarrow \mathrm{Z} \mathrm{b} \overline{\mathrm{b}}$ to the latter. These contributions correspond, respectively, to the regions where the two $\mathrm{b}$ quarks are almost collinear or mostly acollinear. Because two $\mathrm{b}$ jets must be reconstructed, this measurement cannot be sensitive to low-angle gluon splitting, where the distance between the jet-initiating partons is smaller than twice the jet size. This region is better explored by searching directly for pairs of b hadrons close in space, as studied in Ref. [9], whose decay products might be part of a single reconstructed jet. Another angular observable of interest is $\varDelta R_{\mathrm{Z} \mathrm{b}}^{\text {min}}$, the angular separation between the $\mathrm{Z}$ boson and the closer $\mathrm{b}$ jet in the $\eta$–$\phi$ plane. This variable is useful for testing multileg tree-level and NLO corrections in which a $\mathrm{Z}$ boson is radiated from a quark, because it is sensitive to event topologies with the $\mathrm{Z}$ boson in the vicinity of one of the two $\mathrm{b}$ jets. Finally, the $A_{\mathrm{Z} \mathrm{b} \mathrm{b}}$ asymmetry between the $\mathrm{b}$ jet direction and the $\mathrm{Z}$ boson direction is computed using a combination of $\varDelta R_{\mathrm{Z} \mathrm{b}}^{\text {min}}$ and $\varDelta R_{\mathrm{Z} \mathrm{b}}^{\text {max}}$ (the latter being the $\eta$–$\phi$ separation between the $\mathrm{Z}$ boson and the farther b jet):

$$\begin{aligned} A_{\mathrm{Z} \mathrm{b} \mathrm{b}} = \frac{\varDelta R_{\mathrm{Z} \mathrm{b}}^{\text {max}} - \varDelta R_{\mathrm{Z} \mathrm{b}}^{\text {min}}}{\varDelta R_{\mathrm{Z} \mathrm{b}}^{\text {max}} + \varDelta R_{\mathrm{Z} \mathrm{b}}^{\text {min}}}. \end{aligned}$$

The $A_{\mathrm{Z} \mathrm{b} \mathrm{b}}$ asymmetry can provide an indirect test of pQCD validity at higher orders of the perturbative series. A nonzero value of $A_{\mathrm{Z} \mathrm{b} \mathrm{b}}$ is related to the emission of additional gluon radiation in the final state, while values of $A_{\mathrm{Z} \mathrm{b} \mathrm{b}}$ close to zero identify configurations in which the two $\mathrm{b}$ jets are emitted symmetrically with respect to the $\mathrm{Z}$ boson direction.

### Theoretical predictions

The measured differential cross sections for the associated production of $\mathrm{Z}$ bosons and $\mathrm{b}$ jets are compared to several perturbative QCD theoretical calculations.

In pQCD the amplitude for this process can be computed using two alternative approaches. In the four-flavour scheme (4FS) [50], the $\mathrm{b}$ quark mass is explicitly included in the predictions and acts as an infrared cutoff, partly removing possible divergences in the matrix element calculation. This approach corresponds to an effective QCD theory, with $n_{f}=4$ quark flavours involved in the computation of the running of the strong coupling constant $\alpha _S$. In this scheme no $\mathrm{b}$ quark PDF is used, and the $\mathrm{b}$ quark is always produced explicitly by the gluon splitting $\mathrm{g} \rightarrow \mathrm{b} \overline{\mathrm{b}}$ process. In the 5FS [51] (where $n_{f}=5$), the gluon splitting contribution is included within the $\mathrm{b}$ quark PDF, and the $\mathrm{b}$ quark mass is set to zero in the matrix element calculation. The two schemes can be defined in such a way as to provide identical results when all orders in pQCD are computed. However, differences appear in fixed-order predictions, where the different ordering of terms in the matrix element expansion gives different results. The comparison of different flavour schemes is interesting because, in pQCD, the evolution of the $\mathrm{b}$ quark PDF as a function of the Bjorken *x* variable shows sizeable differences between tree-level calculations and those at NLO. These differences are introduced by singularities in the Altarelli–Parisi splitting functions that are present only at NLO; they have no impact on the tree-level evolution of the $\mathrm{b}$ quark PDF [52].

While NLO calculations are now available for both flavour schemes, LO calculations are still interesting to study because they allow the inclusion of multiple additional light, hard partons in the matrix element. This feature is expected to provide a better description of the real hard radiation, compared to fixed-order NLO calculations matched with parton showering.

The MadGraph plus pythia6 event generator, introduced in Sect. 3, describes signal events at full detector simulation level and provides theoretical predictions at tree level for the associated production of $\mathrm{Z}$ bosons and jets, including $\mathrm{b}$ jets. This calculation is based on the 5FS using the LO MadGraph 5.1.3.30 matrix element generator, with up to four additional partons in the matrix element calculation. The factorization and renormalization scales are chosen on an event-by-event basis as the transverse mass of the event, clustered with the $k_{\mathrm {t}}$ algorithm down to a 2$\rightarrow$2 topology, and $k_{\mathrm {t}}$ computed at each vertex splitting, respectively [19, 53]. The matrix element calculation is interfaced with pythia version 6.424, using tune Z2* for parton showering, hadronization, and description of MPI. The CTEQ6L1 PDF is adopted in the calculations. The Drell–Yan inclusive cross section is rescaled to the NNLO calculation provided by fewz 3.1 [20, 21], which has a uncertainty of about 5%. This uncertainty is not propagated into the figures presented below.

Theoretical predictions at tree level based on MadGraph matrix elements for the $\mathrm{Z} + 2\mathrm{b}$ process are also computed using the 4FS MSTW2008 LO PDF set [54]. The factorization and renormalization scales are defined as in the 5FS case. Also in this case, parton showering and hadronization are provided by pythia6 with the tune Z2*. The inclusive cross section is rescaled to the $\mathrm{Z} + 2\mathrm{b}$ NLO calculation with MadGraph5_aMC@NLO [23] for the 4FS, which has an estimated theoretical uncertainty of 15%, dominated by scale variations. This uncertainty is not shown in the figures.

The event generator MadGraph5_aMC@NLO [23] version 2.2.1 is used to provide results at NLO, combining matrix elements for zero, one, and two additional partons through the FxFx algorithm [24]. The NNPDF 3.0 NLO PDF set [26], based on the 5FS, is used. Parton showering and hadronization are performed by pythia version 8.205 [25], using the CUETP8M1 tune [55]. The choice of QCD scales is the same as for the LO MadGraph prediction. This is the same event generator that is used in Sect. 3 to study the systematic uncertainty in the secondary vertex mass distribution.

The 5FS is also used to compute the NLO powheg prediction for a $\mathrm{Z}$ boson associated with two extra partons, including $\mathrm{b}$ quarks [56]. Lower parton multiplicities are described in the matrix element as well, but with no guarantee of NLO accuracy. The scale choice is based on the minlo approach [57]. The NNPDF 3.0 PDF set [26] is used, and the matrix element calculation is matched with the pythia8 parton shower evolution and hadronization, using the CUETP8M1 tune.

For both MadGraph5_aMC@NLO and powheg, no further rescaling of the native cross section is made. Theoretical systematic uncertainties in the predictions, caused by the choice of the QCD factorization and renormalization scales and by the propagation of the uncertainties in PDFs, are computed. The former are estimated by varying the QCD scales by factors of 2 and 0.5, while the latter are computed according to PDF authors’ prescriptions. The uncertainty from varying the QCD scales is generally the dominant contribution. These theoretical uncertainties are displayed in the figures only in the ratio plots, with the statistical uncertainty shown separately, and add up to about 10 and 20% for the two calculations, respectively. For LO calculations, only the statistical uncertainty of theoretical predictions is shown.

### Comparison with data

The measured differential cross sections, unfolded for detector effects, are compatible for the two leptonic channels, and are therefore combined into an uncertainty-weighted average for a single lepton flavour. Correlations between systematic uncertainties for the electron and muon channels are taken into account in the combination. In particular, all uncertainties are treated as fully correlated, with the exception of those related to lepton efficiencies, $\mathrm{t}\overline{\mathrm{t}}$ background estimates, and the statistical part of the subtraction of the c quark and udsg components from $\mathrm{Z}$+jets, and the statistical part of the unfolding uncertainty, which are treated as fully uncorrelated. All the cross sections are measured in the fiducial phase space defined at the generated particle level for the unfolding procedure, as discussed in Sect. 6. No attempt is made to disentangle $\mathrm{b}$ jet production in the hard scattering processes and in MPI.

The integral of the unfolded distributions gives the fiducial cross section, for a single lepton type, for the production of $\mathrm{Z} (1\mathrm{b})$ events,

$$\begin{aligned} \sigma _{\text {fid}}( \mathrm {p}\mathrm {p}\rightarrow \mathrm{Z} +({\ge }1\mathrm{b}) ) = 3.55 \pm 0.12\,\text {(stat)} \pm 0.21\,\text {(syst)} ~\text {pb}, \end{aligned}$$

and $$\mathrm{Z} (2\mathrm{b})$$ events,

$$\begin{aligned} \sigma _{\text {fid}}( \mathrm {p}\mathrm {p}\rightarrow \mathrm{Z} +({\ge }2\mathrm{b})) = 0.331 \pm 0.011\,\text {(stat)} \pm 0.035\,\text {(syst)} ~\text {pb}. \end{aligned}$$

These measured values can be compared with the corresponding predictions at NLO of MadGraph5_aMC@NLO interfaced with pythia8 (described in Sect. 8.2), $4.23^{+0.27}_{-0.37}$ $\text {pb}$ for $\mathrm{Z} (1\mathrm{b})$ and $0.356^{+0.030}_{-0.031}$ $\text {pb}$ for $\mathrm{Z} (2\mathrm{b})$. The prediction overestimates by about 20% the measured value for $\mathrm{Z} (1\mathrm{b})$, while a reasonable agreement is found for $\mathrm{Z} (2\mathrm{b})$ within uncertainties.

The ratio of the cross sections in the fiducial phase space for the production of at least two and at least one $\mathrm{b}$ jet is

$$\begin{aligned} \frac{\sigma _{\text {fid}}( \mathrm {p}\mathrm {p}\rightarrow \mathrm{Z} +({\ge }2\mathrm{b}))}{\sigma _{\text {fid}}( \mathrm {p}\mathrm {p}\rightarrow \mathrm{Z} +({\ge }1\mathrm{b}))} = 0.093 \pm 0.004\,\text {(stat)} \pm 0.007\,\text {(syst)}, \end{aligned}$$

to be compared with the theoretical prediction $0.084^{+0.002}_{-0.001}$ where the systematic uncertainties are considered as fully correlated.

**Fig. 4:**
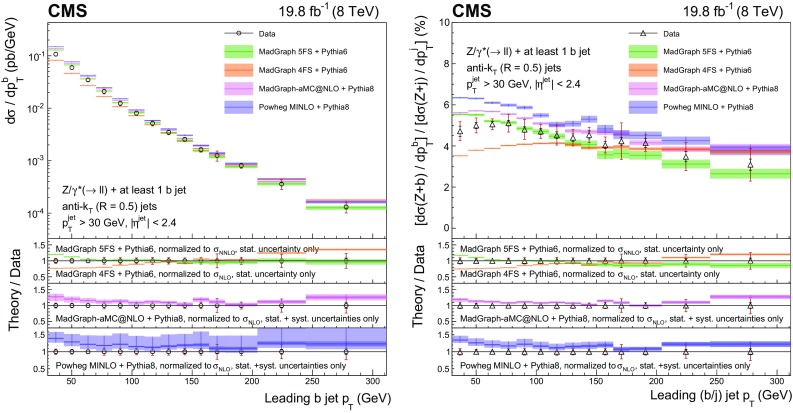
Differential fiducial cross section for Z(1b) production as a function of the leading $\mathrm{b}$ jet $p_{\mathrm {T}}$ (*left*), and the cross section ratio for Z(1b) and Z+jets production as a function of the leading $\mathrm{b}$/inclusive (j) jet $p_{\mathrm {T}}$ (*right*), compared with the MadGraph 5FS, MadGraph 4FS, MadGraph5_aMC@NLO, and powheg minlo theoretical predictions (*shaded bands*), normalized to the theoretical cross sections described in the text. For each data point the statistical and the total (sum in quadrature of statistical and systematic) uncertainties are represented by the *double error bar*. The width of the *shaded bands* represents the uncertainty in the theoretical predictions, and, for NLO calculations, the *inner darker area* represents the statistical component only

**Fig. 5:**
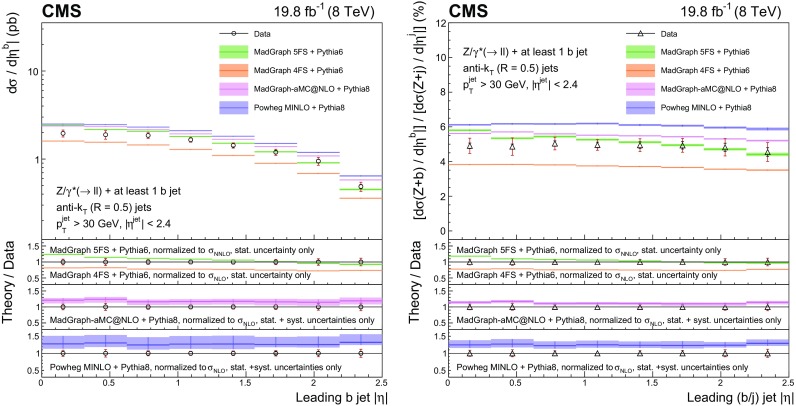
Differential fiducial cross section for Z(1b) production as a function of the leading $\mathrm{b}$ jet $|\eta |$ (*left*), and the cross section ratio for Z(1b) and Z+jets production as a function of the leading $\mathrm{b}$/inclusive (j) jet $|\eta |$ (*right*), compared with the MadGraph 5FS, MadGraph 4FS, MadGraph5_aMC@NLO, and powheg minlo theoretical predictions (*shaded bands*), normalized to the theoretical cross sections described in the text. For each data point the statistical and the total (sum in quadrature of statistical and systematic) uncertainties are represented by the *double error bar*. The width of the *shaded bands* represents the uncertainty in the theoretical predictions, and, for NLO calculations, theoretical systematic uncertainties are added in the ratio plots with the *inner darker area* representing the statistical component only

**Fig. 6:**
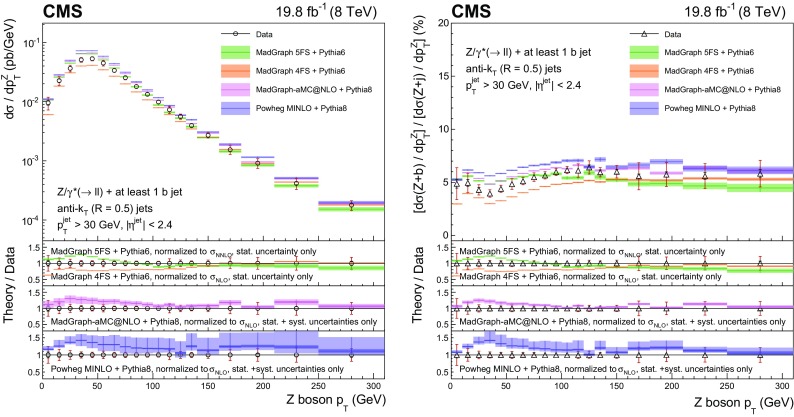
Differential fiducial cross section for Z(1b) production as a function of the $\mathrm{Z}$ boson $p_{\mathrm {T}}$ (*left*), and the cross section ratio for Z(1b) and Z+jets production as a function of the $\mathrm{Z}$ boson $p_{\mathrm {T}}$ (*right*), compared with the MadGraph 5FS, MadGraph 4FS, MadGraph5_aMC@NLO, and powheg minlo theoretical predictions (*shaded bands*), normalized to the theoretical cross sections described in the text. For each data point the statistical and the total (sum in quadrature of statistical and systematic) uncertainties are represented by the *double error bar*. The width of the *shaded bands* represents the uncertainty in the theoretical predictions, and, for NLO calculations, theoretical systematic uncertainties are added in the ratio plots with the *inner darker area* representing the statistical component only

**Fig. 7:**
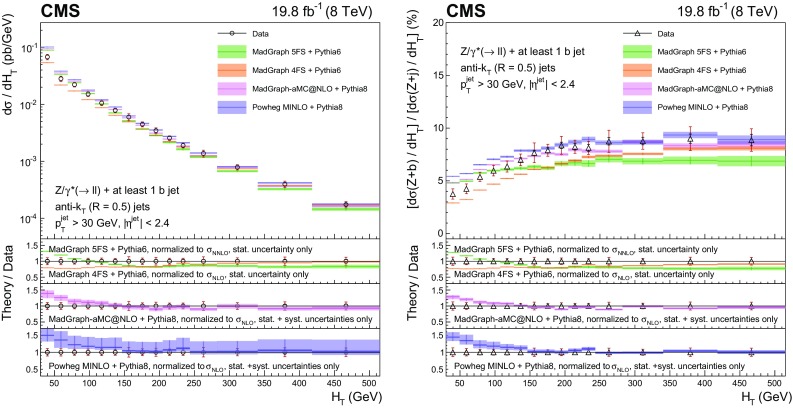
Differential fiducial cross section for Z(1b) production as a function of $H_{\mathrm {T}}$ (*left*), and the cross section ratio for Z(1b) and Z+jets production as a function of $H_{\mathrm {T}}$ (*right*), compared with the MadGraph 5FS, MadGraph 4FS, MadGraph5_aMC@NLO, and powheg minlo theoretical predictions (*shaded bands*), normalized to the theoretical cross sections described in the text. For each data point the statistical and the total (sum in quadrature of statistical and systematic) uncertainties are represented by the *double error bar*. The width of the *shaded bands* represents the uncertainty in the theoretical predictions, and, for NLO calculations, theoretical systematic uncertainties are added in the ratio plots with the inner darker area representing the statistical component only

**Fig. 8:**
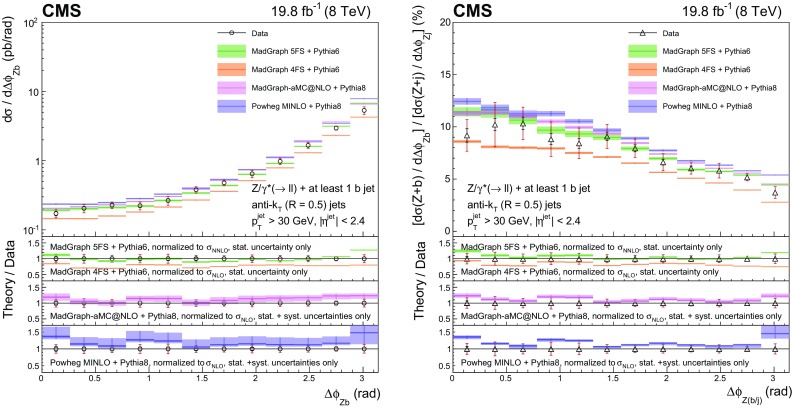
Differential fiducial cross section for Z(1b) production as a function of $\varDelta \phi _{\mathrm{Z} \mathrm{b}}$ (*left*), and the cross section ratio for Z(1b) and Z+jets production as a function of $\varDelta \phi _{\mathrm {Z(b/j)}}$ (*right*), compared with the MadGraph 5FS, MadGraph 4FS, MadGraph5_aMC@NLO, and powheg minlo theoretical predictions (*shaded bands*), normalized to the theoretical cross sections described in the text. For each data point the statistical and the total (sum in quadrature of statistical and systematic) uncertainties are represented by the *double error bar*. The width of the *shaded bands* represents the uncertainty in the theoretical predictions, and, for NLO calculations, theoretical systematic uncertainties are added in the ratio plots with the *inner darker area* representing the statistical component only

Results for the differential cross sections for the $\mathrm{Z} (1\mathrm{b})$ events are presented in Figs. 4, 5, 6, 7 and 8, together with the ratios to the corresponding observables for the inclusive $\mathrm{Z}$+jets event selection. Where applicable, the last bin also includes overflow values. A discrepancy of about 20% is seen in the overall normalization for the 4FS-based prediction, of the same order of magnitude as its estimated theoretical uncertainty. The powheg prediction tends to overestimate the cross sections by about 25%.

Apart for the normalization difference, the pQCD calculation with massive $\mathrm{b}$ quarks (4FS) seems to reproduce, slightly better, the shape of the observed distributions in the soft momentum regime of $\mathrm{b}$ jets. For the leading $\mathrm{b}$ jet $p_{\mathrm {T}}$ spectrum (Fig. 4), the ratio with data is reasonably flat below 80$~\text {GeV}$, whereas it presents a clear slope in the higher $p_{\mathrm {T}}$ range. A similar behaviour is clear in the $\mathrm{Z}$ boson $p_{\mathrm {T}}$ distribution below 130$~\text {GeV}$ (Fig. 6) and in the $H_{\mathrm {T}}$ spectrum below 200$~\text {GeV}$ (Fig. 7). The powheg generator considerably overestimates the soft parts of the $p_{\mathrm {T}}$ and $H_{\mathrm {T}}$ spectra. The leading $\mathrm{b}$ jet $|\eta |$ spectrum shape is well reproduced by the MadGraph 4FS configuration (Fig. 5), while MadGraph 5FS calculation slightly overestimates the central part of the spectrum. The shape of the distribution of the azimuthal angular separation $\varDelta \phi _{\mathrm{Z} \mathrm{b}}$ between the $\mathrm{Z}$ boson and the leading $\mathrm{b}$ jet is reproduced within uncertainties by all the calculations (Fig. 8). The NLO MadGraph5_aMC@NLO predictions have similar behaviour to those from LO MadGraph 5FS. As far as the NLO powheg-based prediction is concerned, it shows a similar behaviour to MadGraph5_aMC@NLO, but the discrepancies are larger, reaching about 40% at the peak of the $\mathrm{Z}$ boson $p_{\mathrm {T}}$ spectrum. In general, the shape predicted by each calculation compares with data, within uncertainties, in a similar way in the high $\mathrm{Z}$ boson $p_{\mathrm {T}}$ and $H_{\mathrm {T}}$ regions, which are potentially sensitive to new physics contributions.

The underestimation of the normalization by MadGraph 4FS and the overestimation by powheg are also observed in the ratio of $\mathrm{Z} (1\mathrm{b})$ and inclusive $\mathrm{Z}$+jets processes (described by the MadGraph generator in the 5FS). The pseudorapidity distribution (Fig. 5), with an almost flat shape, clearly shows that the ratio for the 4FS-based prediction is about 4%, compared to the 5% of the 5FS-based calculations, while powheg predicts about 6%. The 4FS prediction also fails to reproduce the ratio of the leading jet $p_{\mathrm {T}}$ spectra (Fig. 4), which is clearly underestimated below 80$~\text {GeV}$. In contrast, powheg overestimates the spectrum in the soft region by about 30%. Similar discrepancies, although less pronounced, are observed for $H_{\mathrm {T}}$ and the $\mathrm{Z}$ boson $p_{\mathrm {T}}$. The ratio as a function of the azimuthal separation between the $\mathrm{Z}$ boson and the $\mathrm{b}$ jet (Fig. 8) is also slightly underestimated by the MadGraph 4FS prediction when the $\mathrm{Z}$ boson is approximately back-to-back with respect to the leading $\mathrm{b}$ jet, with a difference in the azimuthal angles close to $\pi$.

**Fig. 9:**
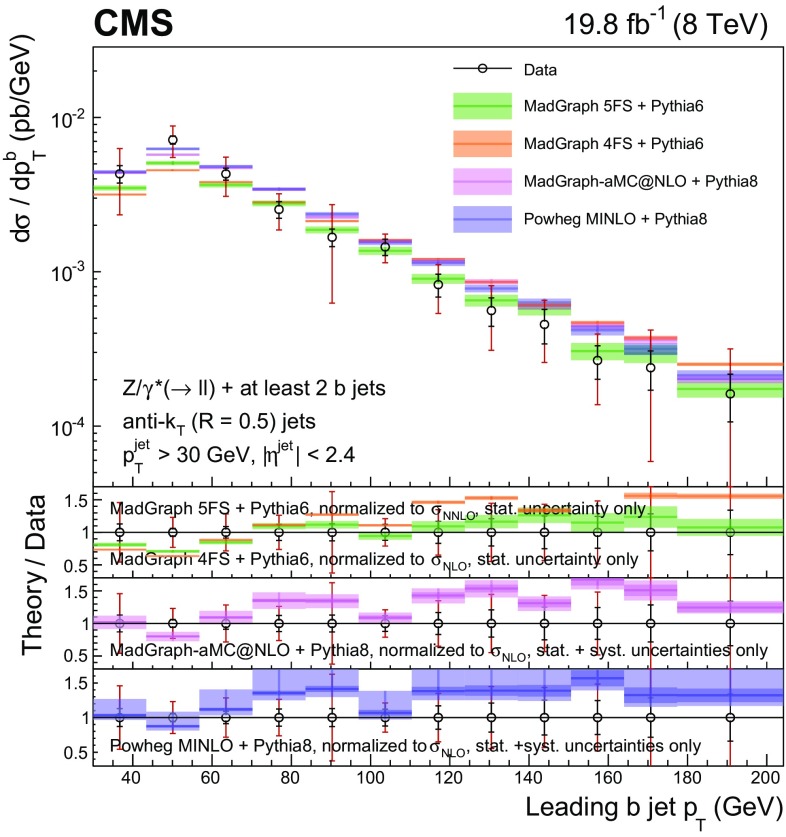
Differential fiducial cross section for Z(2b) production as a function of the leading $\mathrm{b}$ jet $p_{\mathrm {T}}$, compared with the MadGraph 5FS, MadGraph 4FS, MadGraph5_aMC@NLO, and powheg minlo theoretical predictions (*shaded bands*), normalized to the theoretical cross sections described in the text. For each data point the statistical and the total (sum in quadrature of statistical and systematic) uncertainties are represented by the *double error bar*. The width of the *shaded bands* represents the uncertainty in the theoretical predictions, and, for NLO calculations, theoretical systematic uncertainties are added in the ratio plots with the *inner darker area* representing the statistical component only

**Fig. 10:**
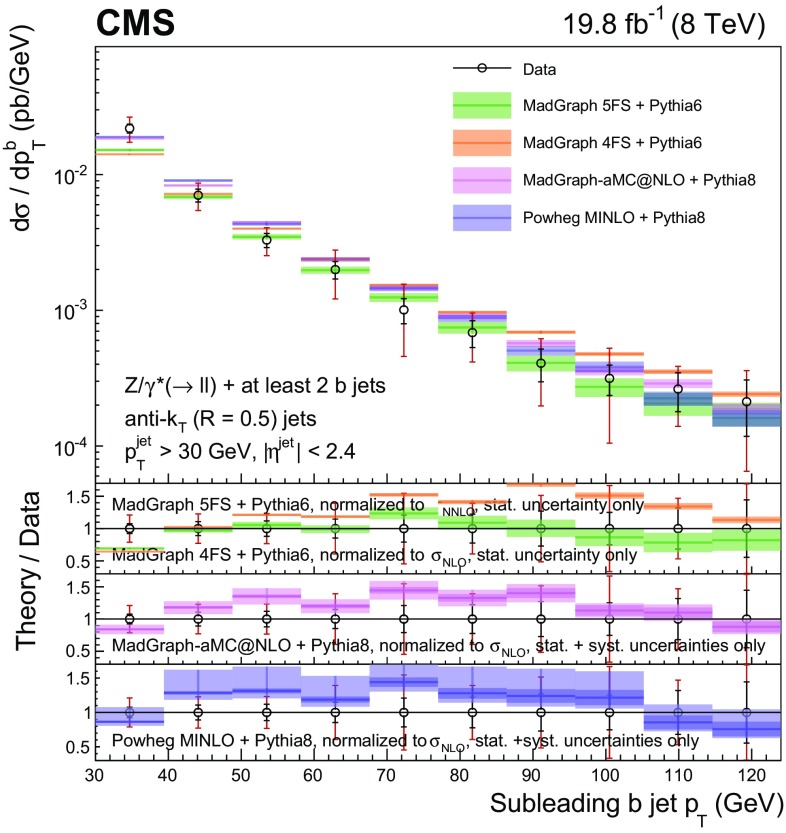
Differential fiducial cross section for Z(2b) production as a function of the subleading $\mathrm{b}$ jet $p_{\mathrm {T}}$, compared with the MadGraph 5FS, MadGraph 4FS, MadGraph5_aMC@NLO, and powheg minlo theoretical predictions (*shaded bands*), normalized to the theoretical cross sections described in the text. For each data point the statistical and the total (sum in quadrature of statistical and systematic) uncertainties are represented by the double error bar. The width of the *shaded bands* represents the uncertainty in the theoretical predictions, and, for NLO calculations, theoretical systematic uncertainties are added in the ratio plots with the *inner darker area* representing the statistical component only

**Fig. 11:**
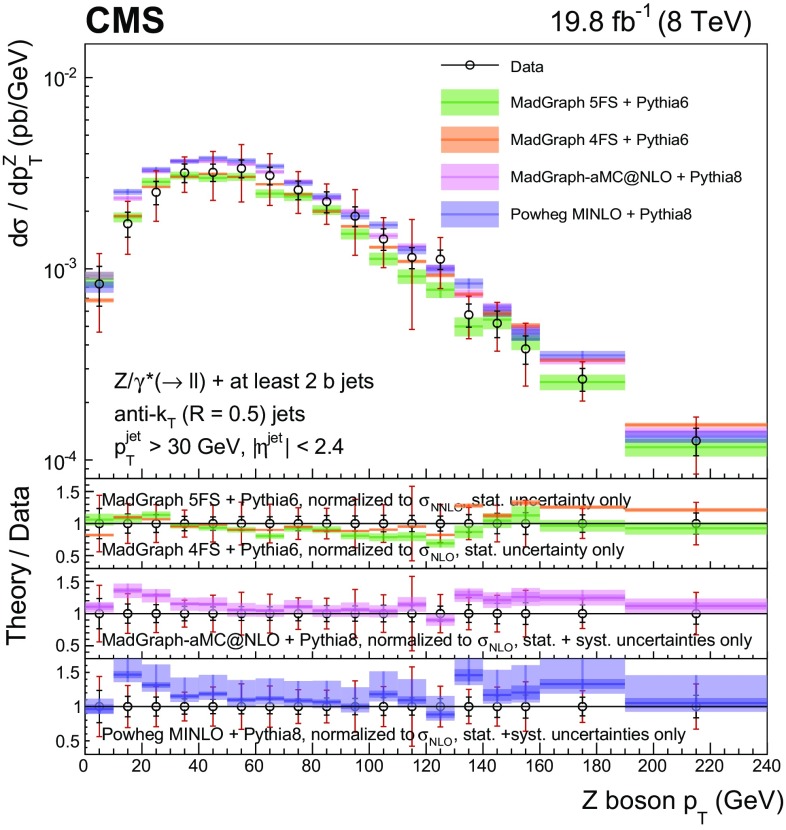
Differential fiducial cross section for Z(2b) production as a function of the $\mathrm{Z}$ boson $p_{\mathrm {T}}$, compared with the MadGraph 5FS, MadGraph 4FS, MadGraph5_aMC@NLO, and powheg minlo theoretical predictions (*shaded bands*), normalized to the theoretical cross sections described in the text. For each data point the statistical and the total (sum in quadrature of statistical and systematic) uncertainties are represented by the *double error bar*. The width of the *shaded bands* represents the uncertainty in the theoretical predictions, and, for NLO calculations, theoretical systematic uncertainties are added in the ratio plots with the *inner darker area* representing the statistical component only

**Fig. 12:**
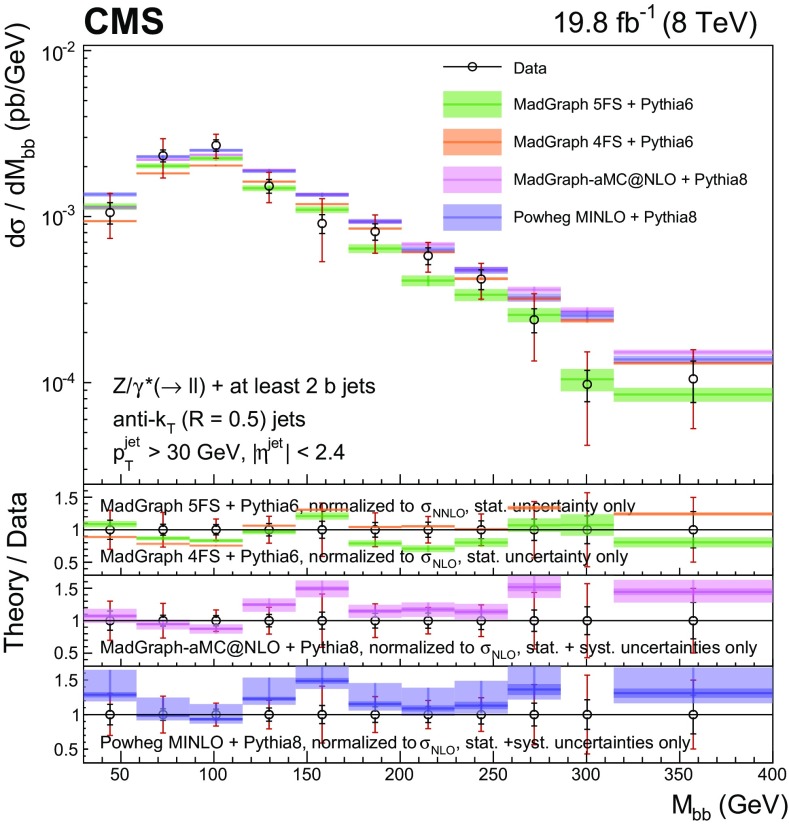
Differential fiducial cross section for Z(2b) production as a function of the invariant mass of the $\mathrm{b}$ jet pair, $M_{\mathrm{b} \mathrm{b}}$, compared with the MadGraph 5FS, MadGraph 4FS, MadGraph5_aMC@NLO, and powheg minlo theoretical predictions (*shaded bands*), normalized to the theoretical cross sections described in the text. For each data point the statistical and the total (sum in quadrature of statistical and systematic) uncertainties are represented by the *double error bar*. The width of the *shaded bands* represents the uncertainty in the theoretical predictions, and, for NLO calculations, theoretical systematic uncertainties are added in the ratio plots with the *inner darker area* representing the statistical component only

**Fig. 13:**
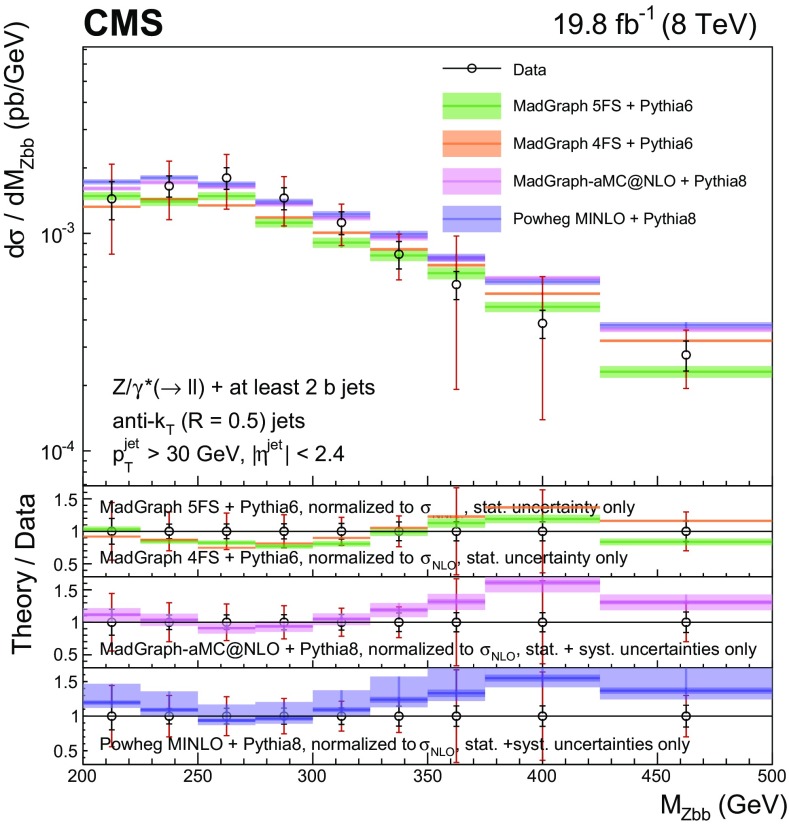
Differential fiducial cross section for Z(2b) production as a function of the invariant mass of the $M_\mathrm{Zbb}$ system, $M_\mathrm{Z} \mathrm{b} \mathrm{b}$, compared with the MadGraph 5FS, MadGraph 4FS, MadGraph5_aMC@NLO, and powheg minlo theoretical predictions (*shaded bands*), normalized to the theoretical cross sections described in the text. For each data point the statistical and the total (sum in quadrature of statistical and systematic) uncertainties are represented by the *double error bar*. The width of the *shaded bands* represents the uncertainty in the theoretical predictions, and, for NLO calculations, theoretical systematic uncertainties are added in the ratio plots with the *inner darker area* representing the statistical component only

**Fig. 14:**
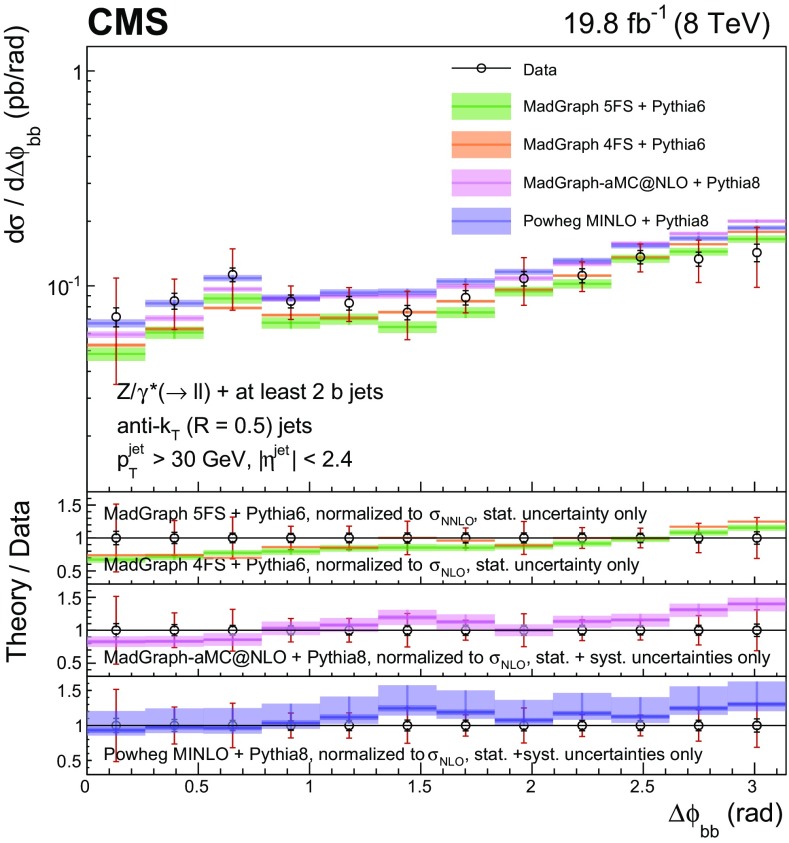
Differential fiducial cross section for Z(2b) production as a function of $\varDelta \phi _{\mathrm{b} \mathrm{b}}$, compared with the MadGraph 5FS, MadGraph 4FS, MadGraph5_aMC@NLO, and powheg minlo theoretical predictions (*shaded bands*), normalized to the theoretical cross sections described in the text. For each data point the statistical and the total (sum in quadrature of statistical and systematic) uncertainties are represented by the *double error bar*. The width of the *shaded bands* represents the uncertainty in the theoretical predictions, and, for NLO calculations, theoretical systematic uncertainties are added in the ratio plots with the *inner darker area* representing the statistical component only

**Fig. 15:**
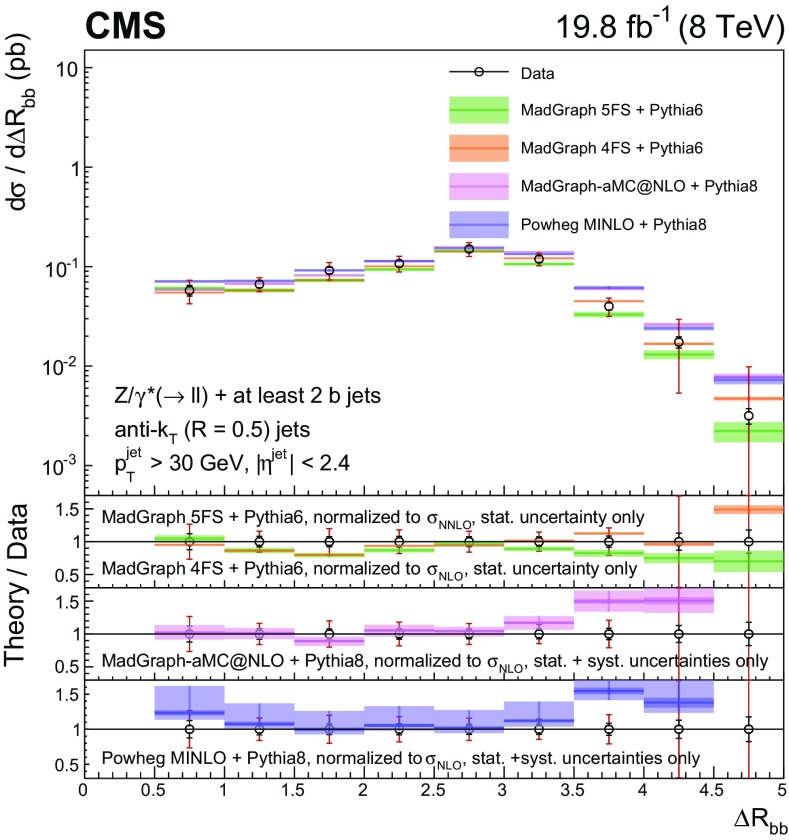
Differential fiducial cross section for Z(2b) production as a function of $\varDelta R_{\mathrm{b} \mathrm{b}}$, compared with the MadGraph 5FS, MadGraph 4FS, MadGraph5_aMC@NLO, and powheg minlo theoretical predictions (*shaded bands*), normalized to the theoretical cross sections described in the text. For each data point the statistical and the total (sum in quadrature of statistical and systematic) uncertainties are represented by the *double error bar*. The width of the *shaded bands* represents the uncertainty in the theoretical predictions, and, for NLO calculations, theoretical systematic uncertainties are added in the ratio plots with the *inner darker area* representing the statistical component only

**Fig. 16:**
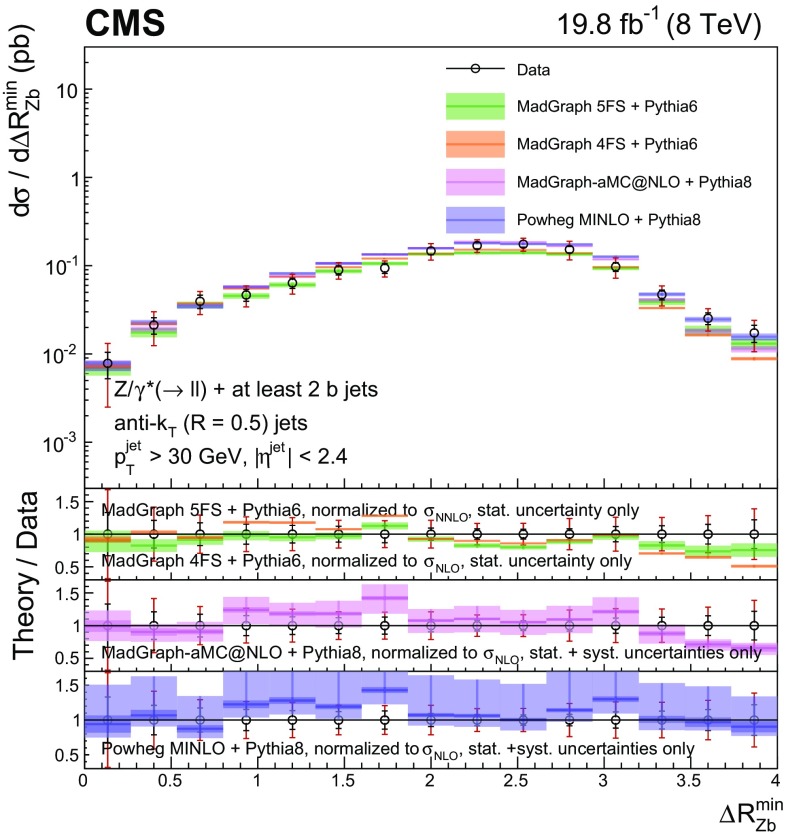
Differential fiducial cross section for Z(2b) production as a function of $\Delta R_\mathrm{Zb}^\text{min}$, compared with the MadGraph 5FS, MadGraph 4FS, MadGraph5_aMC@NLO, and powheg minlo theoretical predictions (*shaded bands*), normalized to the theoretical cross sections described in the text. For each data point the statistical and the total (sum in quadrature of statistical and systematic) uncertainties are represented by the *double error bar*. The width of the *shaded bands* represents the uncertainty in the theoretical predictions, and, for NLO calculations, theoretical systematic uncertainties are added in the ratio plots with the *inner darker area* representing the statistical component only

**Fig. 17:**
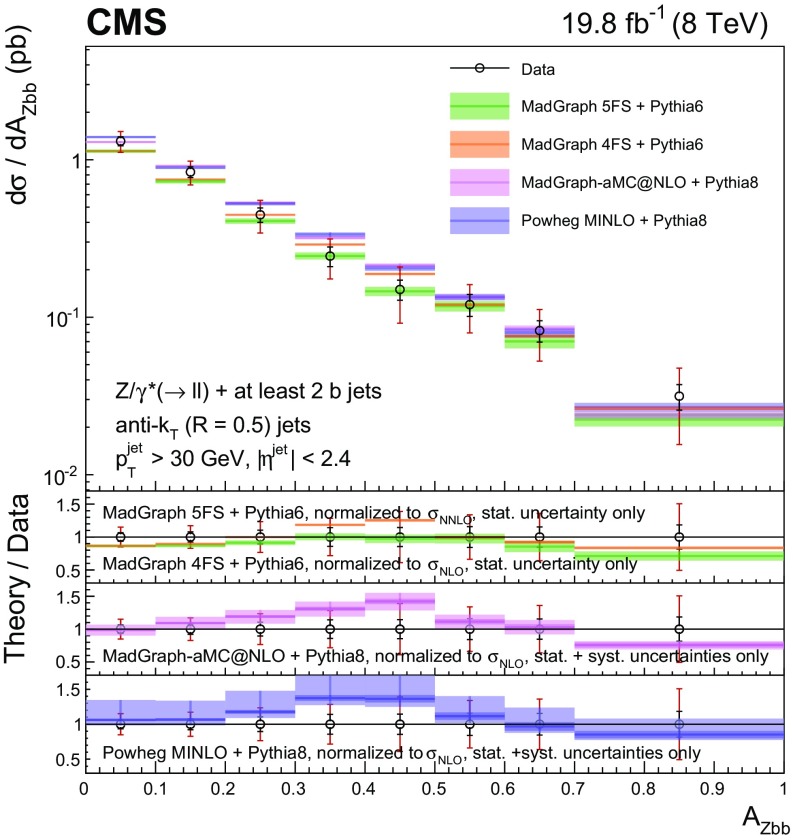
Differential fiducial cross section for Z(2b) production as a function of $A_\mathrm{Zbb}$, compared with the MadGraph 5FS, MadGraph 4FS, MadGraph5_aMC@NLO, and powheg minlo theoretical predictions (*shaded bands*), normalized to the theoretical cross sections described in the text. For each data point the statistical and the total (sum in quadrature of statistical and systematic) uncertainties are represented by the *double error bar*. The width of the *shaded bands* represents the uncertainty in the theoretical predictions, and, for NLO calculations, theoretical systematic uncertainties are added in the ratio plots with the *inner darker area* representing the statistical component only

The results for the differential cross sections measured with the $\mathrm{Z} (2\mathrm{b})$ event selection are shown in Figs. 9, 10, 11, 12, 13, 14, 15, 16 and 17. Within uncertainties, no global normalization discrepancy is observed, contrary to the $\mathrm{Z} (1\mathrm{b})$ case. The leading and subleading jet spectra are slightly underestimated in the soft region by the LO calculations (the leading b jet $p_{\mathrm {T}}$ in the first two bins of Fig. 9 and the subleading b jet $p_{\mathrm {T}}$ in the first bin of Fig. 10), while the $\mathrm{Z}$ boson $p_{\mathrm {T}}$ distribution is well reproduced, within uncertainties (Fig. 11). The 4FS predictions overestimate the data at the high end of these $p_{\mathrm {T}}$ distributions. The ratios of all theoretical predictions and the data show a slight positive slope for the azimuthal separation (Fig. 14). All the other distributions are well reproduced. In general, given the experimental uncertainties, the measurements do not strongly discriminate between the theoretical predictions. The ratio of the MadGraph5_aMC@NLO and powheg predictions based on NLO matrix elements with data shows a similar shape.

## Summary

The process of associated production of jets, including $\mathrm{b}$ jets, and a $\mathrm{Z}$ boson decaying into lepton pairs ($\ell =\mathrm {e},\mu$) are measured in LHC $\mathrm {p}\mathrm {p}$ collisions at $\sqrt{s} = 8\,\text {TeV}$ with the CMS experiment, using a data set corresponding to an integrated luminosity of 19.8$\,\text {fb}^{-1}$. The measured fiducial cross sections are compared to several theoretical predictions. The cross sections are measured as a function of various kinematic observables describing the event topology with a $\mathrm{Z}$ boson and at least one $\mathrm{b}$ jet: the $p_{\mathrm {T}}$ and $\eta$ of the leading $\mathrm{b}$ jet, the $\mathrm{Z}$ boson $p_{\mathrm {T}}$, the scalar sum $H_{\mathrm {T}}$ of the jet transverse momenta, and the azimuthal angular difference between the directions of the leading $\mathrm{b}$ jet and the $\mathrm{Z}$ boson. A comparison is made of the unfolded data with leading-order pQCD predictions based on matrix element calculations matched with parton showering, testing models using the MadGraph event generator, or with the NLO calculations, merging predictions for zero, one, and two extra jets with MadGraph5_aMC@NLO, or for the first two jets with powheg in the minlo approach. In most cases the theoretical predictions agree with the data, although the normalization for MadGraph 4FS, which fails to describe simultaneously both the low- and high-$p_{\mathrm {T}}$ $\mathrm{b}$ jet regions, is underestimated by 20%. The ratios of differential cross sections for the production of a $\mathrm{Z}$ boson in association with at least one $\mathrm{b}$ jet and the inclusive $\mathrm{Z}$+jets production are measured and compared with theoretical expectations. The 4FS-based prediction fails to describe the shape of the ratio as a function of the leading $\mathrm{b}$ jet $p_{\mathrm {T}}$, and discrepancies in the shape are also observed for high values of the $\mathrm{Z}$ boson $p_{\mathrm {T}}$.

The production of a $\mathrm{Z}$ boson in association with two $\mathrm{b}$ jets is also investigated. In this case the kinematic observables are the transverse momenta of the leading and subleading $\mathrm{b}$ jets, the $p_{\mathrm {T}}$ of the $\mathrm{Z}$ boson, the separations of the $\mathrm{b}$ jets both in azimuthal angle and in the $\eta$–$\phi$ plane, the minimal distance in the $\eta$–$\phi$ plane between the $\mathrm{Z}$ boson and a $\mathrm{b}$ jet, the asymmetry between the minimal and the maximal distances between the $\mathrm{Z}$ boson and a $\mathrm{b}$ jet, and the invariant masses of the $\mathrm{b} \mathrm{b}$ and the $\mathrm{Z} \mathrm{b} \mathrm{b}$ systems. The measured distributions are generally well reproduced by the predictions.
